# The Molecular Mechanism of Transport by the Mitochondrial ADP/ATP Carrier

**DOI:** 10.1016/j.cell.2018.11.025

**Published:** 2019-01-24

**Authors:** Jonathan J. Ruprecht, Martin S. King, Thomas Zögg, Antoniya A. Aleksandrova, Els Pardon, Paul G. Crichton, Jan Steyaert, Edmund R.S. Kunji

**Affiliations:** 1 MRC Mitochondrial Biology Unit, University of Cambridge, Cambridge Biomedical Campus, Cambridge CB2 0XY, UK; 2 VIB-VUB Center for Structural Biology, VIB, Pleinlaan 2, 1050 Brussels, Belgium; 3 Structural Biology Brussels, Vrije Universiteit Brussel, Pleinlaan 2, 1050 Brussels, Belgium; 4 Computational Structural Biology Section, National Institute of Neurological Disorders and Stroke, NIH, Bethesda, MD 20892, USA

**Keywords:** adenine nucleotide translocator, adenine nucleotide translocase, bongkrekate, cardiolipin, induced fit, transport mechanism, bioenergetics, mitochondria, alternating access mechanism

## Abstract

Mitochondrial ADP/ATP carriers transport ADP into the mitochondrial matrix for ATP synthesis, and ATP out to fuel the cell, by cycling between cytoplasmic-open and matrix-open states. The structure of the cytoplasmic-open state is known, but it has proved difficult to understand the transport mechanism in the absence of a structure in the matrix-open state. Here, we describe the structure of the matrix-open state locked by bongkrekic acid bound in the ADP/ATP-binding site at the bottom of the central cavity. The cytoplasmic side of the carrier is closed by conserved hydrophobic residues, and a salt bridge network, braced by tyrosines. Glycine and small amino acid residues allow close-packing of helices on the matrix side. Uniquely, the carrier switches between states by rotation of its three domains about a fulcrum provided by the substrate-binding site. Because these features are highly conserved, this mechanism is likely to apply to the whole mitochondrial carrier family.

**Video Abstract:**

## Highlights

• Structure of the matrix-open state of the mitochondrial ADP/ATP carrier solved
• The inhibitor bongkrekic acid locks the state by occupying the substrate-binding site
• Conformational changes during transport are highly dynamic, using six mobile elements
• Roles of all conserved sequence features in mitochondrial carriers are now explained

## Graphical Abstract

**Figure:**
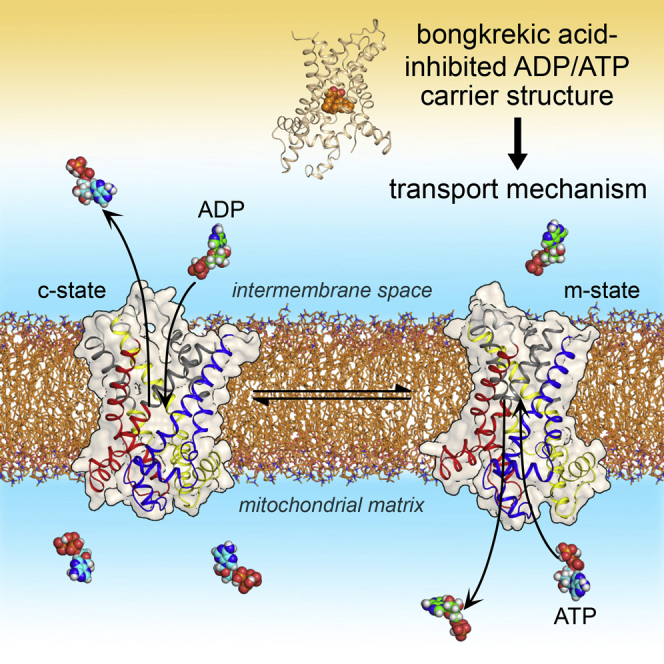

## Teaser

The structure of the matrix-open state of the mitochondrial ADP/ATP carrier reveals its transport mechanism, which involves rotation of six structural elements around a central substrate-binding site.

## Introduction

Solute transport across the impermeable mitochondrial inner membrane is essential for the function and survival of eukaryotic cells. The majority of these transport steps are catalyzed by membrane proteins of the mitochondrial carrier family (Monné and Palmieri, 2014). The archetypal member of the family, the ADP/ATP carrier, performs the vital role of transporting ADP into the mitochondrial matrix and ATP out of the mitochondrion to maintain high cytosolic ATP concentrations for energy-requiring reactions (Kunji et al., 2016). Every day, this carrier transports our own body weight in ADP and ATP, recycling each ATP molecule more than a thousand times. The carrier cycles between the cytoplasmic-open state (c-state), which can be trapped by the membrane-impermeable toxic inhibitor carboxyatractyloside (CATR) (Vignais et al., 1973), and the matrix-open state (m-state), which is locked by the membrane-permeable bongkrekic acid (BKA) (Erdelt et al., 1972, Henderson and Lardy, 1970). BKA is a polyunsaturated methoxy tricarboxylic acid polyketide produced by *Burkholderia gladioli* pathological variant *cocovenenans* (de Bruijn et al., 1973, van Veen and Mertens, 1934). Over 2,000 cases of human fatality from BKA poisoning have been reported in Indonesia, China, and Mozambique since 1950, due to contamination of corn or coconut products (*tempe bongkrek*) (Anwar et al., 2017, Falconer et al., 2017).

Mitochondrial carriers consist of three homologous sequence repeats of about 100 amino acids (Saraste and Walker, 1982), which form a 3-fold pseudosymmetrical structure with the translocation path through the center of the molecule (Kunji and Harding, 2003). The first atomic structure of the CATR-inhibited bovine ADP/ATP carrier (Pebay-Peyroula et al., 2003) was followed by those of yeast ADP/ATP carriers in the same state (Ruprecht et al., 2014). Each sequence repeat forms a domain, comprising an odd-numbered transmembrane helix (H1, H3, or H5), a loop containing a short matrix helix lying parallel to the membrane (h12, h34, or h56), and an even-numbered transmembrane helix (H2, H4, or H6) (Pebay-Peyroula et al., 2003). In the C-terminal region of each odd-numbered transmembrane helix, a conserved signature motif Px[DE]xx[KR] is found (Pebay-Peyroula et al., 2003). The proline residues of this motif are located at pronounced kinks in the odd-numbered helices, giving them an L-shape. Consequently, these helices come together on the matrix side, where the charged residues of the three motifs form the matrix salt bridge network, closing the central cavity to the mitochondrial matrix in the c-state (Pebay-Peyroula et al., 2003).

Nucleotides are among the largest solutes to cross biological membranes, yet mitochondrial carrier proteins are relatively small. Transport must therefore involve profound conformational changes, which nevertheless must prevent proton leak across the mitochondrial inner membrane. Until now, the molecular mechanism by which mitochondrial carriers transport has not been established. However, some sequence features required for a transport mechanism have been identified. First, a consensus substrate-binding site has been proposed in the central cavity (Dehez et al., 2008, Robinson and Kunji, 2006, Robinson et al., 2008, Wang and Tajkhorshid, 2008). Second, a conserved motif [YF][DE]xx[KR] was identified on the even-numbered helices, the charged residues of which likely form a cytoplasmic salt bridge network in the m-state (King et al., 2016, Robinson et al., 2008, Ruprecht et al., 2014). It has been proposed that substrate binding leads to the interconversion of states by disruption and formation of the cytoplasmic and matrix salt bridge networks, opening and closing the carrier to either side of the membrane (Kunji et al., 2016, Robinson and Kunji, 2006, Ruprecht et al., 2014). Here, we have determined the structure of the ADP/ATP carrier inhibited by BKA, which represents the first structure of a carrier in an m-state conformation and reveals the molecular mechanism of transport utilized by mitochondrial carrier proteins.

## Results

### A Nanobody-Stabilized BKA-Inhibited ADP/ATP Carrier Structure

**Figure S1:**
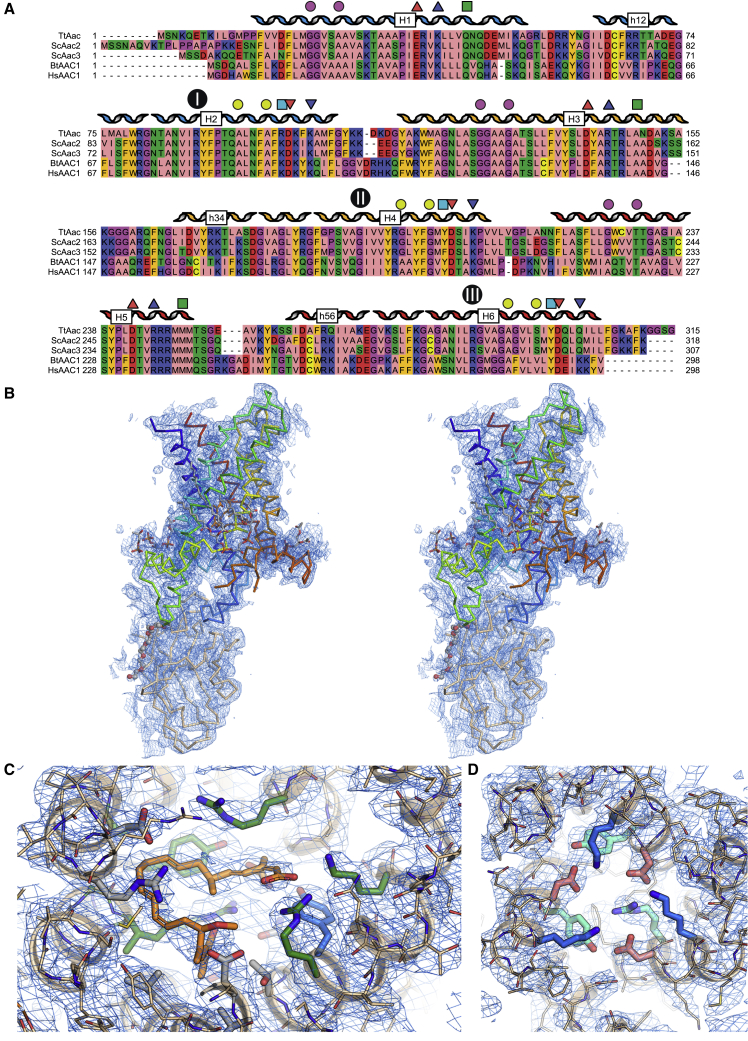
Alignment of the Amino Acid Sequences of Selected Mitochondrial ADP/ATP Carriers and Representative Electron Density of the TtAac-Nb Complex, Related to Figure 1 (A) Alignment of the mitochondrial ADP/ATP carriers from *Thermothelomyces thermophila* (TtAac), from *Saccharomyces cerevisiae* isoform 2 (ScAac2) and isoform 3 (ScAac3), and bovine (BtAAC1) and human (HsAAC1) isoform 1. Amino acids are colored according to their properties: basic K, R and H are blue, acidic D and E are red, polar N, Q, S and T are green, aliphatic A, I, L, M and V are pink, aromatic F, Y and W are orange, structural G and P are magenta, and C is yellow. The negatively charged (red) and positively charged (blue) residues of the matrix and cytoplasmic networks are indicated by up and down triangles, respectively. The positions of the glutamine brace (Q brace) and tyrosine brace (Y brace) are indicated by green and cyan squares, even if they are not conserved in ADP/ATP carriers. The purple and lime circles indicate the positions of the GxxxG and πxxxπ motifs. The contact points of the substrate binding site are shown in black circles with roman numerals (Robinson and Kunji, 2006). (B) Stereo-view showing the contents of the asymmetric unit, with the carrier shown in rainbow colored ribbon representation, and the nanobody as a wheat ribbon. A PEG molecule is shown in ball-and-stick representation, and partially-modeled cardiolipins as sticks. The blue mesh shows the final 2*m*Fo-*D*Fc electron density map, contoured at 1 times the root mean square electron density, and shown within 5 Å of the atoms. (C) Detailed view of the bongkrekic acid-binding site. BKA is shown with orange carbons. Amino acids that are part of the putative substrate-binding pocket are shown with green carbon atoms. Additional amino acids that form salt bridges or hydrogen bonds, or van der Waals interactions, are shown with blue or gray carbon atoms, respectively. The blue mesh shows the final 2*m*Fo-*D*Fc electron density map, sharpened by applying a negative *B*-factor (*B* = −45 Å^2^), contoured at 1 times the root mean square electron density, and shown within 5 Å of the atoms. (D) Detailed view of the cytoplasmic salt bridge network and brace. The blue mesh shows a different view of the map in (C).

Mitochondrial carriers are highly dynamic proteins, and the m-state is known to be unstable in detergent solution (Crichton et al., 2015), which has hampered structure determination. To solve these problems, we targeted the BKA-inhibited ADP/ATP carrier from the moderately thermophilic fungus *Thermothelomyces thermophila* (TtAac, Figure S1), carrying a single mutation (Q302K) in the cytoplasmic network that increases the thermal stability (King et al., 2016). Furthermore, we selected a nanobody against this state and crystallized the complex (see STAR Methods for details). TtAac has 75% sequence identity to *Saccharomyces cerevisiae* ScAac2 and ScAac3 and 51% sequence identity to bovine Aac1p, the structures of which have been determined in the CATR-inhibited state (Pebay-Peyroula et al., 2003, Ruprecht et al., 2014). Crystals diffracted anisotropically to 3.3 Å (Table S1, TtAac-Nb crystal), enabling structure determination (Figure S1).

**Figure S2:**
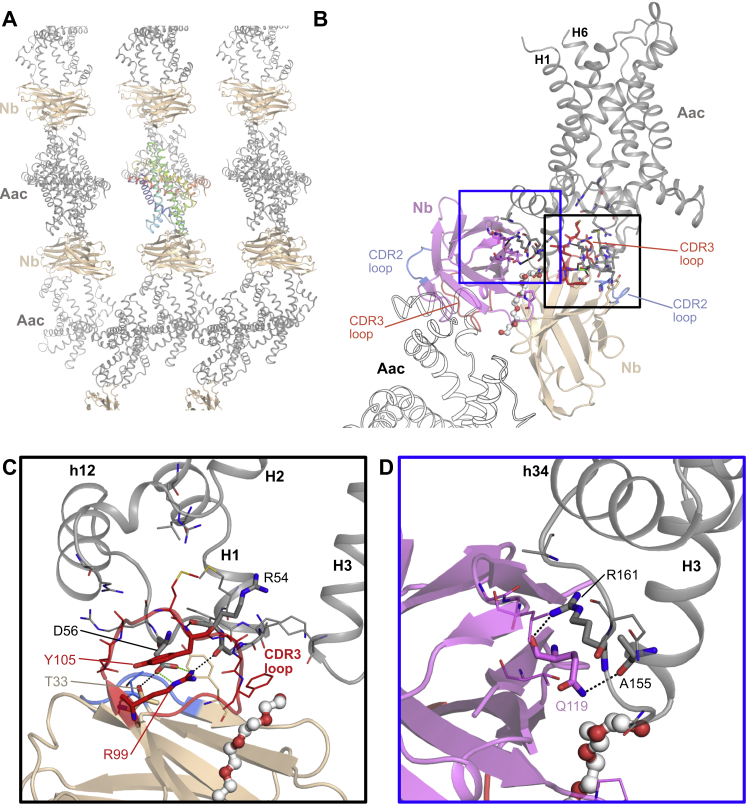
Interactions between Nanobody and ADP/ATP Carrier, Related to Figure 1 (A) Crystal packing shows alternating layers of carrier proteins (Aac, gray cartoon, with one molecule highlighted as a rainbow cartoon) and nanobodies (Nb, wheat cartoon), with the nanobodies playing key roles in forming crystal contacts. (B) In the crystal, each ADP/ATP carrier molecule (Aac, gray cartoon) interacts with one nanobody (wheat cartoon, interactions highlighted in black box) via the nanobody CDR2 (blue) and CDR3 (red) loops, and with a symmetry-related nanobody (violet cartoon, interactions highlighted in blue box). (C) An enlarged view of the interactions involving the nanobody CDR2 and 3 loops. The CDR3 loop interacts with the matrix end of H1, the matrix helix h12, and the loop connecting them, via hydrogen bond and electrostatic interactions (between D56 of the carrier and R99 of the nanobody). (D) An enlarged view of the interactions with a symmetry-related nanobody (violet cartoon), with A155 and R161 of the carrier forming hydrogen bonds with Q119. These interactions are likely to be solely due to crystal packing.

**Figure 1:**
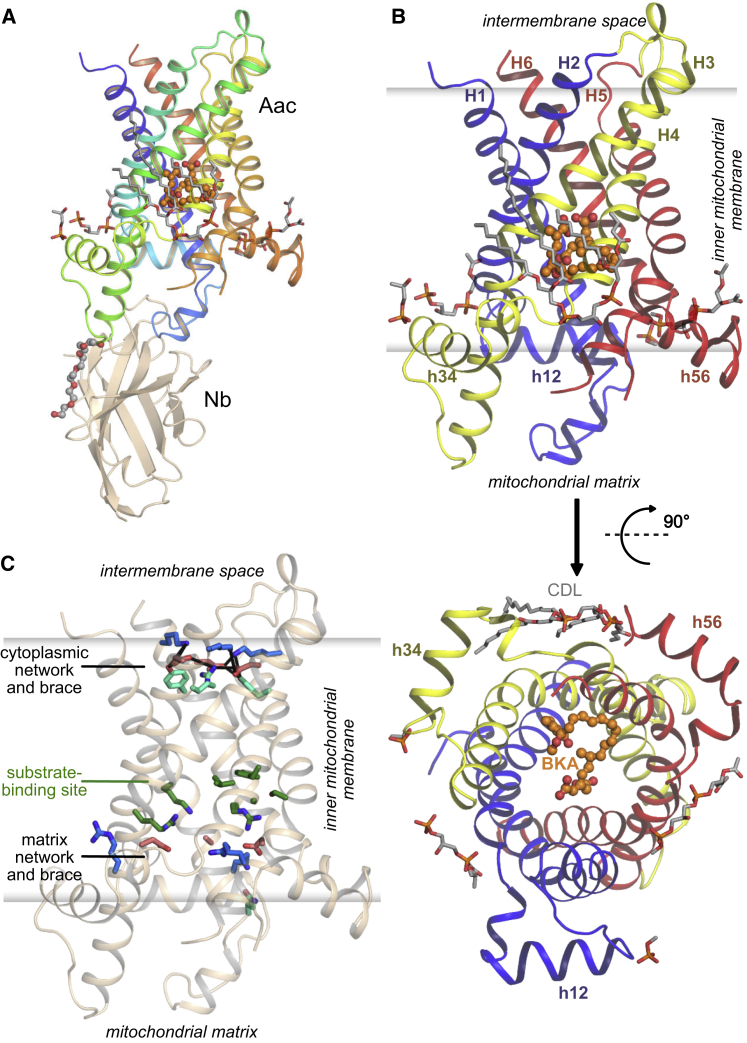
Overall Structure of the Bongkrekic Acid-Inhibited ADP/ATP Carrier (A) The structure of TtAac (rainbow cartoon) bound to bongkrekic acid (ball-and-stick with orange carbons) and a nanobody (Nb, wheat cartoon). Cardiolipin molecules associated with the carrier are shown as sticks (gray carbons) and a PEG molecule is shown in ball-and-stick representation (gray carbons). (B) The carrier protein viewed from the membrane (top), with domains 1, 2, and 3 colored in blue, yellow, and red, respectively, and from the mitochondrial matrix side (bottom). Transmembrane helices (H1 to H6) and matrix helices (h12, h34, h56) are labeled. (C) The BKA-inhibited carrier viewed from the membrane with key functional elements highlighted. Residues of the substrate-binding site are shown as sticks with green carbons. Positively charged (blue carbons) and negatively charged (red carbons) residues of the cytoplasmic and matrix networks are also shown, along with their braces (cyan carbons). See also Figures S1–S5 and Table S1.

**Figure S3:**
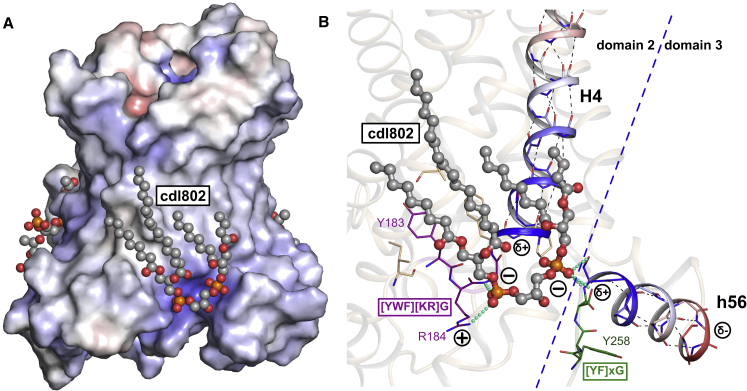
Bound Cardiolipin Acts as an Inter-domain Bridge, Related to Figures 1 and 6 (A) Cardiolipin binding sites (ball-and-stick representation, with gray carbon atoms) with cdl802 highlighted. The protein is shown in surface representation, colored by electrostatic potential (blue, +10 kT e^-1^; white, neutral; red, −10 kT e^-1^). Cardiolipin phosphates occupy pockets with positive electrostatic potential. (B) Detailed view of the binding site for cdl802. Cardiolipin phosphate groups form hydrogen bonds with the amide groups at the N-terminal ends of the even-numbered and matrix helices, and interact with the positively-charged ends of the helix dipoles, as seen in the c-state (Ruprecht et al., 2014). The cardiolipin molecule is shown in ball-and-sticks. Amino acids making interactions with the lipid are shown as thin sticks. Residues in the conserved [YWF][RK]G and [YF]xG motifs are shown in purple and green, respectively. Hydrogen bond and electrostatic interactions between protein and lipid are shown by cyan dashed lines. The helix dipoles associated with the even-numbered and matrix helices are shown (blue, δ+, to red, δ-), and intra-helical hydrogen bonds are shown as thin dashed black lines.

The crystal is composed of alternating layers of carrier and nanobody molecules, with crystal contacts between the carrier layers being mediated by the nanobodies (Figure S2). The carrier consists of six transmembrane helices surrounding a central cavity (Figure 1). The structural fold has the characteristic three-domain structure of this protein family (Figure 1B). The matrix salt-bridge network (E37, K40, D142, R145, D242, and R245) and cytoplasmic salt-bridge network (D101, K104, D205, K208, D299, and K302) flank the proposed central substrate-binding site (K30, R88, G192, I193, Y196, S238, and R287) (Figure 1C). The carrier binds one molecule of BKA and several cardiolipin molecules, which bind to positively charged pockets due to the N-terminal dipoles of the matrix and even-numbered transmembrane helices (Figure S3).

**Figure 2:**
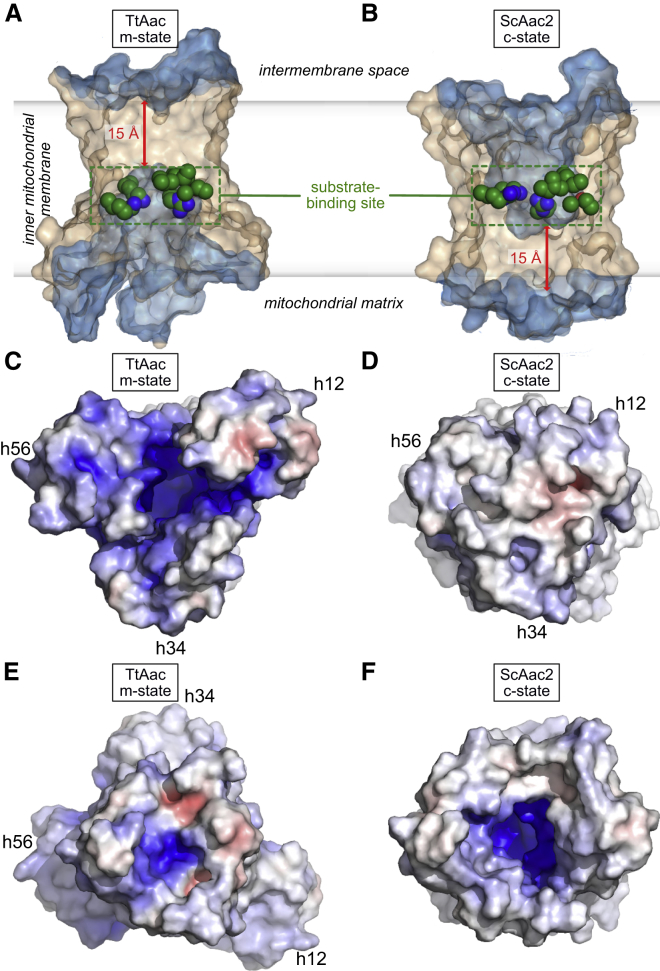
Alternating Access in the ADP/ATP Carrier (A and B) Surface view of the BKA-inhibited carrier (A; m-state) and of the CATR-inhibited *S. cerevisiae* Aac2p carrier (B; c-state, PDB: 4C9H). The blue semi-transparent surfaces reveal the internal cavities. The residues of the substrate-binding site (spheres with green carbons) lie at the center of the membrane and are alternately accessible to the mitochondrial matrix (BKA-inhibited carrier) or cytoplasmic (CATR-inhibited carrier) sides of the membrane. (C and D) The BKA-inhibited carrier (C) and CATR-inhibited carrier (D) viewed from the mitochondrial matrix. (E and F) The BKA-inhibited carrier (E) and the CATR-inhibited carrier (F) viewed from the cytoplasmic side of the membrane. They are shown as a surface colored by electrostatic potential (blue, +15 kT e^−1^; white, neutral; red, −15 kT e^−1^). The positions of the matrix helices are indicated in (C)–(E). See also Figures S4 and S5.

**Figure S4:**
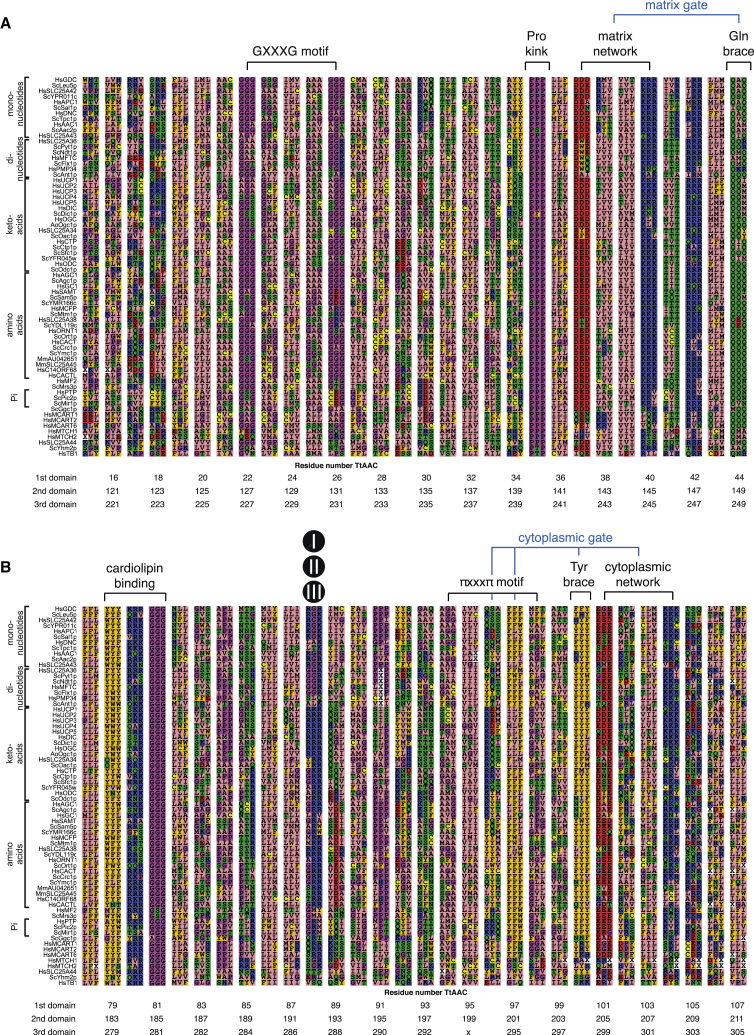
Alignment of Symmetry-Related Triplets from Yeast and Human Carrier Sequences, Related to Figures 1, 2, 3, 5, and 6 (A) The odd-numbered helices and (B) even-numbered helices of different mitochondrial carriers of *Saccharomyces cerevisiae* (Sc), *Homo sapiens* (Hs), *Musculus musculus* (Mm), and *Aspergillus oryzae* (Ao). The residues are shown as a triplet of symmetry-related residues of domain 1, 2 and 3 together to emphasize the symmetry in the three-fold repeats of mitochondrial carriers (Robinson et al., 2008). Below the triplets are the corresponding numbers of the residues in TtAac that form the symmetry-related triplet. Amino acids are colored according to their properties as in Figure S1. Indicated above are the functional elements of mitochondrial carriers and the contact points of the substrate binding site in black circles with roman numerals (Robinson and Kunji, 2006). Figure adapted from Figures S1 and S3 from Robinson et al. (2008). Copyright (2008) National Academy of Sciences.

**Figure S5:**
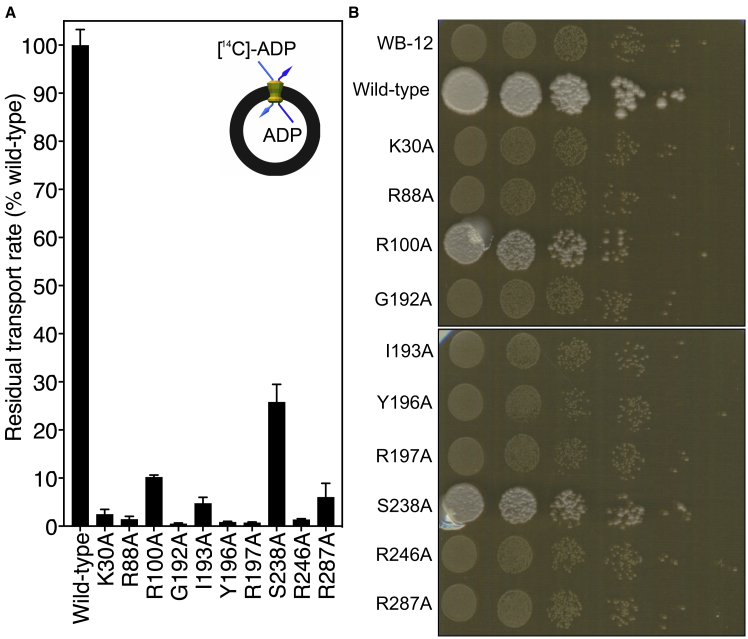
Transport Activity and Growth on Glycerol of Alanine Replacement Mutants of the Substrate-Binding Site Residues of TtAac, Related to Figures 1, 2, and 4 (A) Transport assays in fused membrane vesicles of *Lactococcus lactis*, expressing the wild-type and single alanine replacement mutants of TtAac. (B) Drop test on YPG plates of yeast expressing wild-type and single alanine replacement mutants of TtAac, showing that none of the binding site mutants are capable of growing on glycerol. R100A serves as a control.

Comparison of the BKA-inhibited carrier structure with the CATR-inhibited c-state structure of the homologous *S. cerevisiae* ADP/ATP carrier Aac2p (ScAac2, PDB: 4C9H) reveals a profound conformational change (Figure 2). The BKA-inhibited m-state has a central cavity open to the mitochondrial matrix side of the membrane, with the matrix helices rotated outward (Figures 1 and 2). In the m-state, the cavity is closed to the cytoplasmic side of the membrane by a cluster of residues that includes those of the cytoplasmic salt bridge network (Figures 1C and S4). In the c-state, which is open to the cytoplasmic side, the cytoplasmic network residues are far apart, whereas the matrix salt bridge network residues are interacting, helping to close the carrier on the matrix side (Pebay-Peyroula et al., 2003, Ruprecht et al., 2014). Solvent accessibility calculations map out the geometry of the central cavity in both states and reveal that each state has an ∼15 Å thick gate, closing off access to the central cavity from one side of the membrane and thus preventing proton leak (Figures 2A and 2B). The consensus residues of the proposed substrate-binding site (Kunji et al., 2016), which we confirm are essential for the function of TtAac (Figure S5), are accessible for substrate binding in both states. This observation provides structural support for the single-binding center gated pore hypothesis (Klingenberg, 1979), which is essentially an alternating access model (Jardetzky, 1966). Comparing surface representations shows the dramatically different shapes of the two states (Figures 2C–2F). Viewed from the matrix side, the m-state has a strong positively charged central cavity around the substrate-binding site, primed for binding negatively charged ATP (Figure 2C), whereas access to the cavity is closed in the c-state with a predominantly neutral surface (Figure 2D). Viewed from the intermembrane space, the cavity is closed in the m-state, with a predominantly neutral surface, except for a small positively charged patch (Figure 2E), whereas a positively charged cavity is visible in the c-state (Figure 2F).

### A Gate Blocks Access from the Cytoplasmic Side

**Figure 3:**
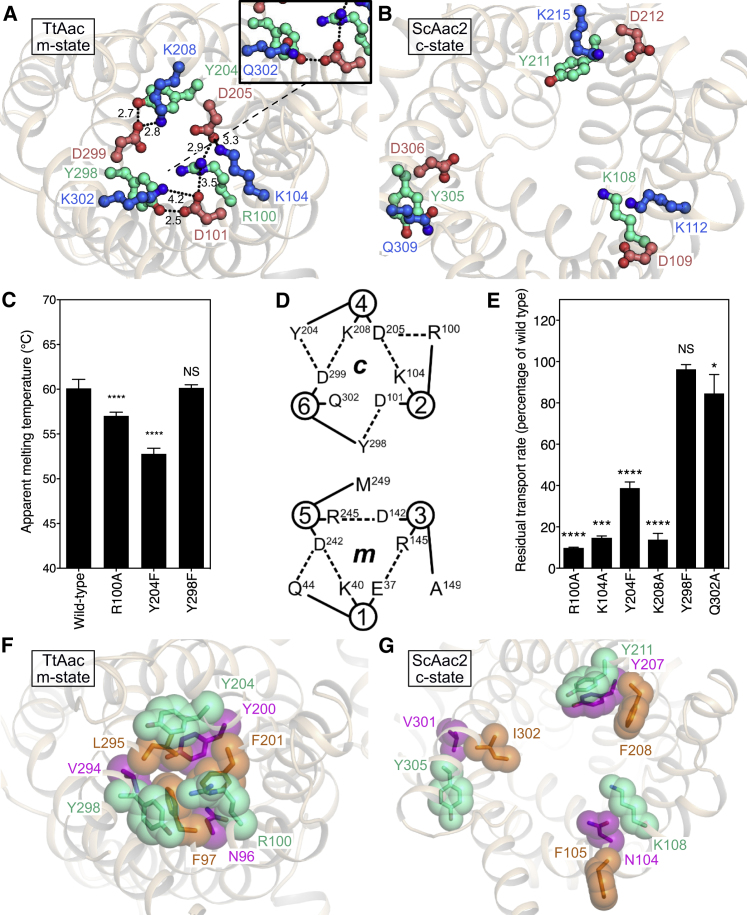
Cytoplasmic Salt Bridge Network and Gate (A) Cytoplasmic salt bridge network and tyrosine braces in the BKA-inhibited carrier, showing polar interactions (black dotted lines) with indicated distances (Å). The inset shows interactions in the wild-type carrier. (B) View of the cytoplasmic salt bridge network and brace in CATR-inhibited carrier (ScAac2, PDB: 4C9H). (A) and (B) are viewed from the cytoplasmic side of the membrane. (C) Mutation of R100 and Y204 residues bracing the cytoplasmic network reduces the thermal stability of BKA-inhibited carriers. The apparent melting temperature is represented by the mean ± SD, with n = 6 for mutants and n = 12 for wild-type. (D) Schematic diagram illustrating the cytoplasmic (*c*) and matrix (*m*) networks in TtAac. The circles represent transmembrane helices, with numbers indicating the corresponding helices. Polar inter-domain interactions are marked by black dashes. (E) The residual transport rates of brace mutants, represented by the mean ± SD, with n = 4. (F) The cytoplasmic gate in the BKA-inhibited carrier. (G) Cytoplasmic gate in the CATR-inhibited carrier. (F) and (G) are viewed from the cytoplasmic side of the membrane. For (C) and (E), p > 0.05, not-significant (NS); ^∗^p > 0.01, ^∗∗∗^p > 0.0001, and ^∗∗∗∗^p > 0.00001 by two-tailed Student’s t test. See also Figure S4 and Table S2.

The structure reveals that in the m-state, the charged residues of the cytoplasmic salt bridge network, part of the conserved [YF][DE]xx[KR] motif (Figure S4), come into close proximity and form electrostatic interactions, linking the even-numbered transmembrane helices of each domain (Figure 3A) in agreement with functional data (King et al., 2016). K104 on H2 forms a salt bridge with D205 on H4, and K208 on H4 interacts with D299 on H6. K302 on H6, which was mutated to stabilize the m-state (King et al., 2016), interacts weakly with D101 on H2. In the wild-type carrier, the corresponding residue is Q302, which when modeled *in silico* is too far away to interact with D101 directly (Figure 3A, inset). Close to the salt bridge residues, a highly conserved set of tyrosine residues, positioned a turn of helix away from the positively charged residues of the network, unexpectedly act as braces for the salt bridges, providing extra inter-domain interactions (Figures 3A and S4). Y204 forms a hydrogen bond with D299, bracing the K208-D299 salt bridge. Y298 forms a hydrogen bond with D101, compensating for the weak D101-K302 interaction. We call these extra interactions tyrosine braces. ADP/ATP carriers are unusual in the mitochondrial carrier family in having a positively charged residue substituting for tyrosine on H2 (Figure S4). Here, R100 forms electrostatic interactions with both D101 (intra-domain) and D205, bracing the K104/D205 salt bridge in an equivalent way to the tyrosine braces. These tyrosine braces, which are part of the highly conserved [YF][DE]xx[KR] motif, serve a similar function to the glutamine braces observed for the matrix salt bridge network (Ruprecht et al., 2014). The number of these braces vary in different members of the mitochondrial carrier family, modulating the interaction energy of the networks (Figure S4). Comparison with the c-state shows that the residues have moved 12–17 Å for the formation of the cytoplasmic network (Figure 3B).

We have previously studied the effect of cytoplasmic salt bridge network mutations on the thermal stability of the BKA-inhibited carrier, showing that these interactions contribute significantly to the overall thermal stability of the protein in the matrix state (King et al., 2016). The structure reveals that there are few inter-domain interactions other than those of the cytoplasmic salt-bridge network and brace residues, which is an important functional feature of this highly dynamic protein. Therefore, the relative network strength correlates well with the overall stability of the protein. Here, we extend our analysis to include the tyrosine brace interactions (Figure 3C). In the BKA-inhibited state, mutation of R100 and Y204 significantly reduces the thermal stability compared to the wild-type protein, consistent with these residues forming a brace that stabilizes the m-state. Y298F did not have a significant effect on thermal stability of the BKA-inhibited state compared to the wild-type, consistent with the Y298-D101 bond forming the weakest link in the network.

Previously, the effect of substrate-binding and salt bridge formation and disruption on the transport rate was analyzed by treating the carrier as a nanomachine moving stochastically between the m-state and c-state under the influence of thermal energy (Springett et al., 2017). This computational analysis shows that maximum transport occurs when the interaction energies of the cytoplasmic network, matrix network, and substrate binding are approximately equal. As a consequence, the analysis predicted that interactions in addition to the cytoplasmic salt bridge network should stabilize the matrix state. The current structure reveals that the tyrosine braces provide these additional interactions, resulting in the cytoplasmic and matrix networks having similar numbers of polar interactions (Figure 3D). The effect of mutations of the cytoplasmic salt bridge network and braces on the transport rate was investigated (Figure 3E). Mutations of residues involved in electrostatic interactions (R100, K104, and K208) had the greatest effect on the transport rate, followed by Y204. Mutations of Q302 and Y298 had only a mild effect. Thus, mutations of the strongest links in the cytoplasmic network have the largest effects upon transport and thermal stability, as expected from the structure of the cytoplasmic network in the m-state and the computational model.

Just beneath the tyrosine brace residues, there are other aromatic or bulky hydrophobic residues (F97, F201, and L295) that come together to form a hydrophobic plug. These residues converge when the carrier is in the m-state (Figure 3F) but are separate in the c-state (Figure 3G). The residues of the hydrophobic plug, together with the cytoplasmic salt bridge network and tyrosine braces, form the cytoplasmic gate, which is highly conserved in all members of the mitochondrial carrier family, highlighting their important role in the transport cycle (Figure S4). Another set of residues, which are variable within the mitochondrial carrier family, form the ceiling of the substrate-binding site (N96, Y200, and V294).

### Bongkrekic Acid Is a Conformation-Specific Competitive Inhibitor

**Figure 4:**
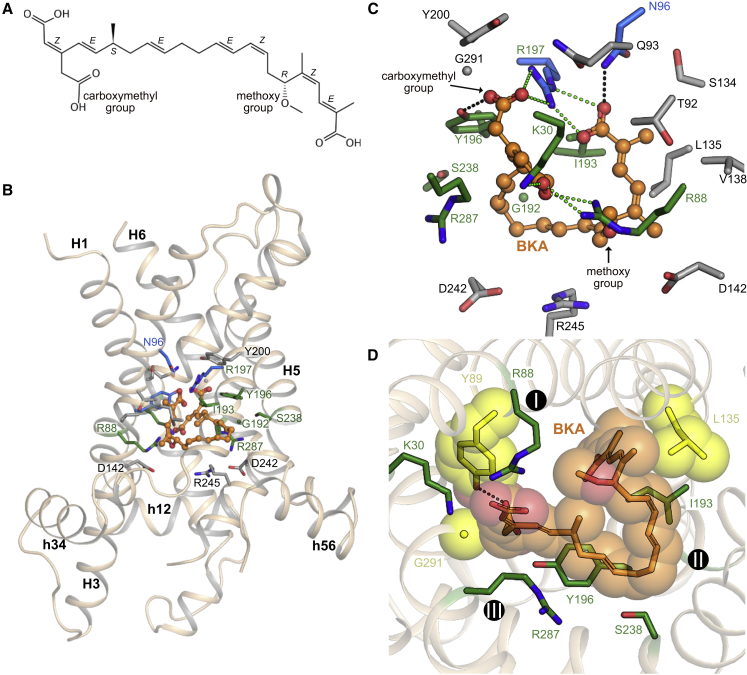
Structural Basis for Inhibition of the ADP/ATP Carrier by Bongkrekic Acid (A) Structure of the inhibitor bongkrekic acid (BKA) with the stereochemistry of chiral centers and double bonds labeled. (B) Overview of the inhibitor-binding site. The carrier is shown as a wheat cartoon, with BKA in ball-and-stick representation (orange carbon atoms). Amino acid residues forming the binding-pocket are shown in stick representation. Amino acids that are part of the putative substrate-binding pocket are shown with green carbon atoms. Additional amino acids that form salt bridges or hydrogen bonds, or van der Waals interactions, are shown with blue or gray carbon atoms, respectively. (C) Detailed view of the BKA-binding site, with salt bridges shown as green dashed lines and hydrogen bonds as black dashed lines. Y89, which forms a hydrogen bond to BKA, has been removed to provide a clearer view. (D) Mutations that confer resistance to BKA (Zeman et al., 2003) affect residues that interact or are in van der Waals contact with BKA (yellow), but do not affect the putative substrate-binding site (green). The contact points of the substrate-binding site are indicated by black spheres with white Roman numerals. The hydrogen bond between Y89 and BKA is indicated by the black dashed line. The spheres indicate the van der Waals radii of the affected residues and BKA. See also Figures S4 and S6.

**Figure S6:**
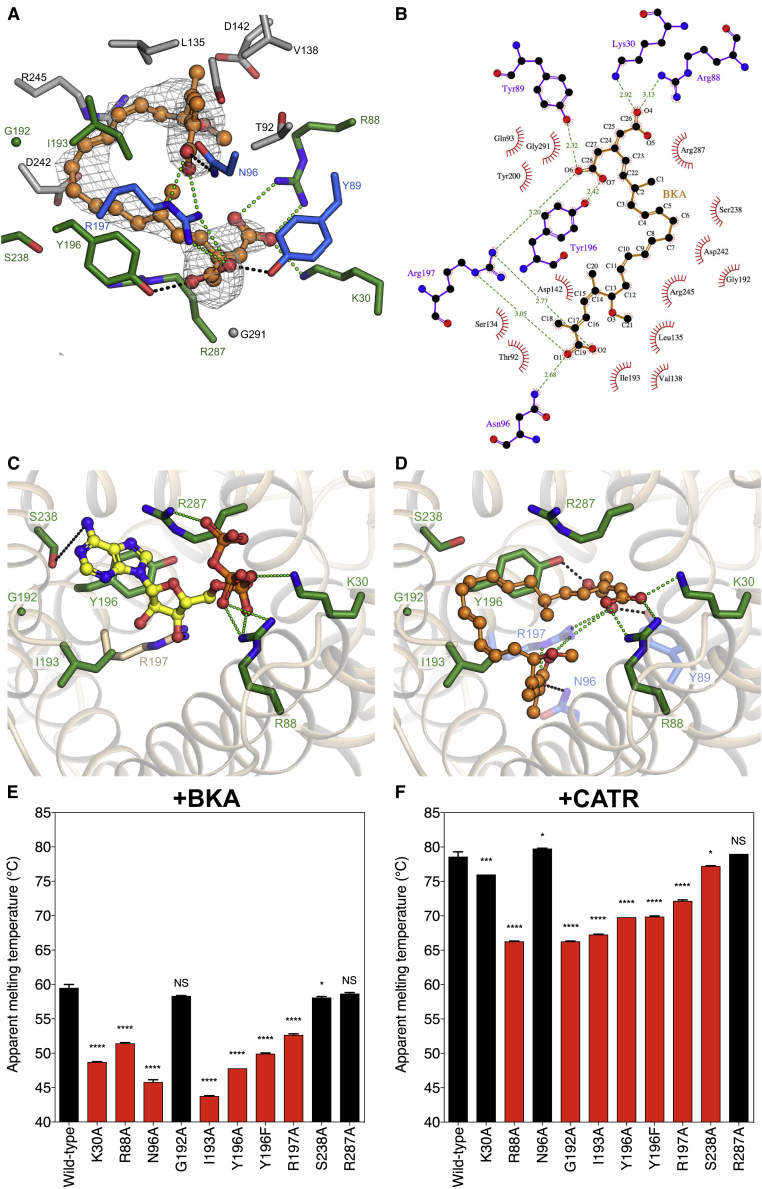
Binding of Bongkrekic Acid and ATP, Related to Figure 4 (A) *m*F_o_-*D*F_c_ polder OMIT electron density map for BKA, contoured at 3 times the root mean square electron density, shown with the final model. Q93 and Y200, which are in hydrophobic contact with BKA, have been removed to provide a clearer view. (B) Schematic drawing of the binding-site, showing amino acid residues within 4 Å of BKA. Hydrogen bonds and salt bridges are shown as green dashed lines with indicated distances (Å). Red arcs indicate residues in hydrophobic contact with the inhibitor, with spokes radiating toward the atoms they contact. Figure generated by Ligplot+ (Laskowski and Swindells, 2011). (C) Putative binding mode of ATP. The model was generated by docking in AutoDock 4.2 (Forli et al., 2016), treating the protein as a rigid molecule and with S238 modeled as an alternative common rotamer. The model shows the adenine ring of ATP binding to a hydrophobic pocket formed by residues Y196 (aromatic stacking), G192 and I193. S238 may form a hydrogen bond to the adenine ring, and R197 a hydrogen bond to the ribose (black dotted lines). The phosphate groups of ATP interact with the positively-charged residues K30, R88 and R287 (green dotted lines). The features of this binding site mimic those predicted for ADP binding to the c-state (Dehez et al., 2008, Kunji and Robinson, 2006, Mifsud et al., 2013, Robinson and Kunji, 2006, Wang and Tajkhorshid, 2008). (D) View of the BKA binding-site from the matrix side, highlighting residues in the putative substrate-binding site (shown in stick representation). Electrostatic interactions between these residues and BKA are shown as dotted green lines, and hydrogen bonds as dotted black lines. The view is the same as (C) illustrating the overlap between the BKA and ATP-binding site. (E) Mutation of binding-site residues reduces the thermal stability of BKA-inhibited variants. Residues forming polar interactions with BKA, or proximal to the inhibitor, are highlighted in red. (F) Binding-site mutants show increased thermal stability in the presence of CATR, hence are folded correctly and able to bind the inhibitor. CATR induces a greater stabilization than BKA. Mutation of residues that form salt bridges to CATR (R88, R197), or form the binding-pocket (G192, I193, Y196, S238) (Ruprecht et al., 2014), reduces the apparent melting temperature of the CATR-inhibited protein. These residues are shown with red bars. Mutation of residues distal to CATR (K30, S238) results in a smaller reduction in apparent melting temperature. In (E) and (F), the apparent melting temperature is represented by the mean ± S.D., with n = 3. p > 0.05, not-significant (NS); p > 0.01, ^∗^; p > 0.0001, ^∗∗∗^; p > 0.00001, ^∗∗∗∗^ by two-tailed Student’s t test.

Electron density maps calculated following molecular replacement revealed extra density in the cavity, not accounted for by protein atoms. As model building and refinement progressed, this density could be modeled as the inhibitor BKA (Figures 1, 4, S1C, and S6A).

BKA inhibits the carrier by binding deeply and asymmetrically within the central cavity (Figures 1 and 4). The polyunsaturated backbone of the inhibitor forms a horseshoe shape, allowing the carboxylate groups at either end of the inhibitor to interact with key amino acids that lie close together in space. BKA interacts directly with all residues of the putative substrate-binding site (Kunji et al., 2016), forming electrostatic interactions with K30 and R88 and a hydrogen bond to Y196 (Figures 4B and 4C). The carboxymethyl and distal carboxylate group at either end of the inhibitor both form electrostatic interactions with R197, and additional hydrogen bonds are formed to N96 and Y89. The large number of interactions formed between the inhibitor and protein explains the tight binding of BKA (Figure S6B) (Klingenberg et al., 1983). ATP can be docked into the binding site (Figure S6C), with the phosphate groups interacting with K30, R88, and R287, and the adenine ring sitting in a hydrophobic pocket formed by G192, I193, Y196, and S238 and potentially forming aromatic stacking interactions with the tyrosine, in agreement with previous work based on the c-state (Kunji et al., 2016). Comparison of the ATP and BKA binding geometries shows that the dicarboxylate end of BKA mimics the phosphate groups of ATP, while the polyunsaturated backbone of the inhibitor acts as a partial mimic of the adenine ring of the substrate, sitting in the hydrophobic pocket (Figures S6C and S6D). The position and steric bulk of the polyunsaturated backbone would prevent the substrate from binding to the m-state. BKA thus shows the characteristics of a conformation-specific competitive inhibitor.

Random mutagenesis of the related ScAac2 has produced mutant strains of yeast that are resistant to BKA but can still grow on non-fermentable carbon sources, which requires the carrier to be functional (Zeman et al., 2003). Four mutations were identified and three of them are within the observed BKA-binding site (Y97C, L142S, and G298S, equivalent to Y89, L135, and G291 in TtAac). These residues are in van der Waals contact with BKA (Figure 4D), and the Y97C mutation would remove a hydrogen bond to BKA. The fourth mutation lies within the GxxxG motif, described below (Cappello et al., 2007). Interestingly, the key residues involved in substrate-binding are not affected by these mutations, explaining why transport could still occur. The agreement between the position of mutations and the BKA-binding site confirms that the observed BKA binding pose reflects the one found in ScAac2 in the native membrane. In another approach, the role of residues near the cavity in binding BKA had been assessed prior to structure determination by comparing the thermostability of the BKA-inhibited wild-type with those of BKA-inhibited mutants (Figure S6E). Mutating key residues involved in BKA-binding (e.g., K30, R88, N96, I193, Y196, and R197) reduced the stabilizing effect of BKA binding observed in the wild-type protein, consistent with weaker binding of the inhibitor. Mutagenesis of R287 or S238, which form weak van der Waals interactions with BKA rather than polar interactions, did not significantly reduce the thermal stability of the inhibited carrier. The thermal stability of the mutants in the presence of CATR confirms that they are folded correctly (Figure S6F). Mutation of residues that are known to interact with CATR in the closely related ScAac2 (Ruprecht et al., 2014), has a strong effect on thermal stability of the CATR-inhibited state, whereas a weaker effect is seen for those that are further away, confirming that thermostability assays can be used to map inhibitor-binding sites.

### GxxxG and πxxxπ Motifs Allow Close Packing of Helices on the Cytoplasmic Side

**Figure 5:**
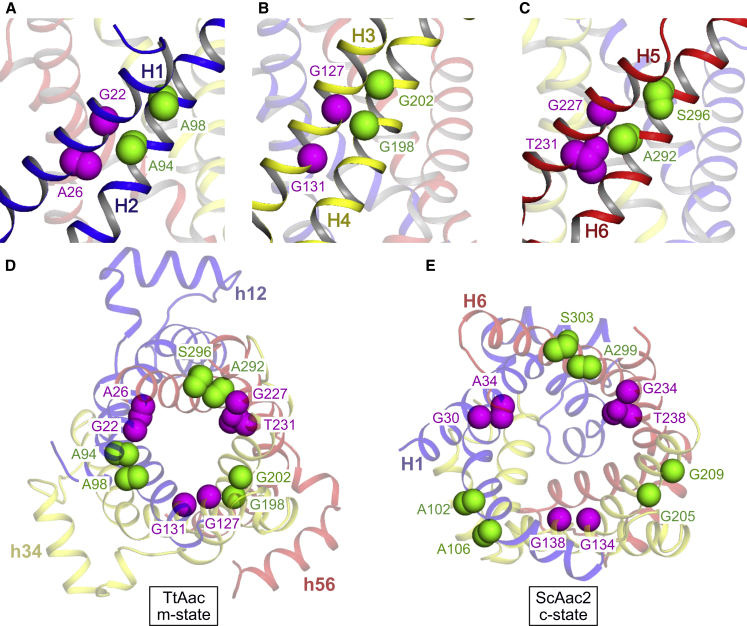
Role of Conserved Glycines and Residues with Small Side Chains (A) Role of conserved small amino acids in the intra-domain interface. Domain 1 is highlighted (blue cartoon). (B and C) Equivalent views for (B) domain 2 (yellow) and (C) domain 3 (red). Residues of the GxxxG motif and the πxxxπ motif are shown as magenta and green spheres, respectively. (D) Overview of residues forming the m-state intra-domain inter-helical interface, viewed from the cytoplasmic side of the membrane. (E) Equivalent residues, shown in the c-state (PDB: 4C9H). In (D) and (E), the carrier is shown in cartoon representation, with domains colored (domain 1, blue; domain 2, yellow; domain 3, red). Residues are shown as spheres, colored as in (A)–(C). See also Figure S4.

In the BKA-inhibited state, transmembrane helices are packed closely on the cytoplasmic side of the membrane, helping to form the cytoplasmic gate. This is facilitated by amino acids with small side chains lying at the intra-domain interfaces, which include residues of the highly conserved GxxxG motif on the odd-numbered helices (Figures 5 and S4) (e.g., G127 and G131 on H3). Other conserved small residues are found on the adjacent even-numbered helices (e.g., G198 and G202 on H4), which we call the πxxxπ motif after the one letter code for small amino acids (Figures 5B and S4). A similar arrangement is seen in domains 1 and 3 (Figures 5A and 5C). The requirement for residues with small side chains at these positions explains their conservation, which was not apparent from the c-state structures (Figures 5D and 5E).

For the inter-domain interfaces, close-packing of helices at the cytoplasmic side is facilitated by conserved small amino acids on the odd-numbered transmembrane helices, for example A130 on H3 of domain 2. Residues on the even-numbered helices do not point directly at the interface but face the lipid bilayer (L95 or F99) or cavity (N96, R100), hence larger side-chains can be tolerated here. A similar arrangement is observed in all three inter-domain interfaces in the m-state structure and also in the c-state structures (Ruprecht et al., 2014).

### Conformational Changes between C- and M-States

**Figure 6:**
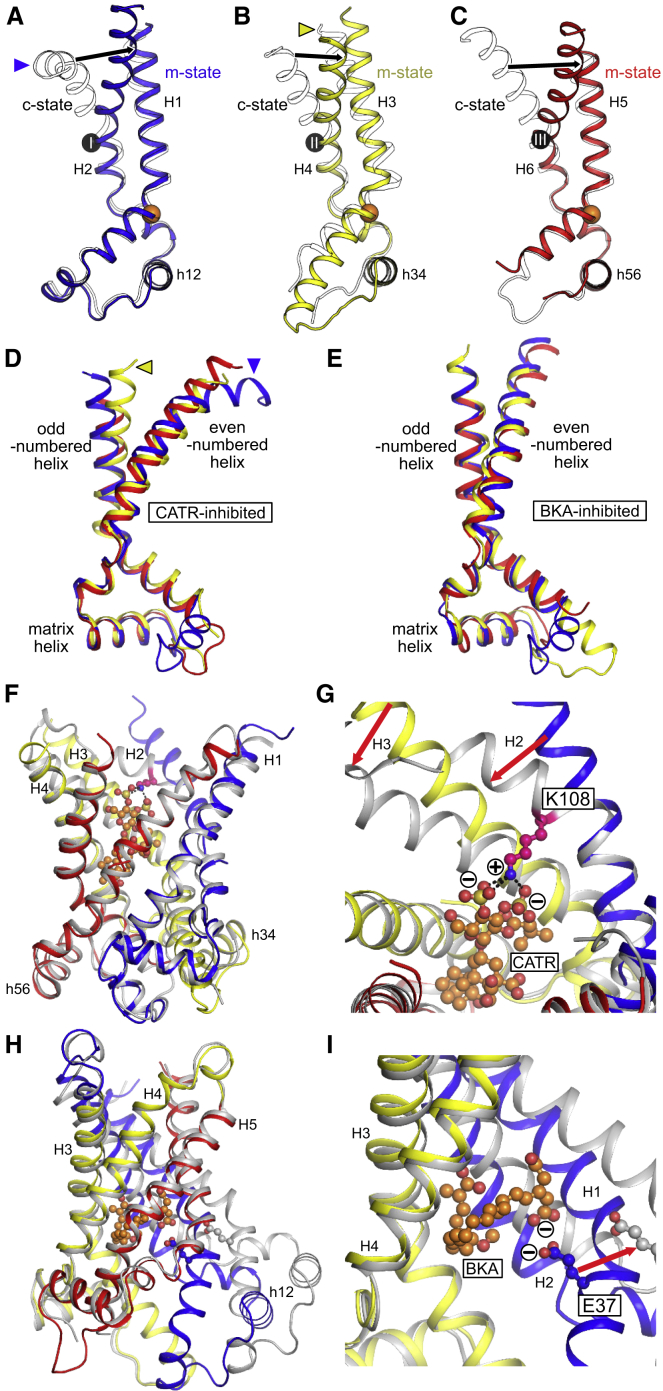
Conformational Changes between M- and C-States and the Structural Consequences of Inhibitor Binding (A) Structure of domain 1 in the m-state (blue cartoon) versus the c-state (PDB: 4C9H, outline). (B and C) Similar views of (B) domain 2 (m-state yellow cartoon, c-state outline) and (C) domain 3 (m-state red cartoon, c-state outline), respectively. The orange spheres mark the positions of the conserved prolines of the signature sequence Px[DE]xx[KR]. The numbered black spheres mark the positions of the substrate-binding site contact points (Robinson and Kunji, 2006). Domains have been aligned on their core elements. (D) Superposition of domains 1 to 3 of CATR-inhibited ScAac2 (PDB: 4C9H, colored by domain, as in A–C). The kink at the cytoplasmic-end of H2 is indicated by a blue triangle and is also highlighted in (A). The yellow triangle highlights the displacement of H3, also apparent in (B). (E) Superposition of domains 1 to 3 of BKA-inhibited TtAac (colored by domain, as in A–C), confirming the conservation of domain structure. (F) Superposition of gate and core elements from the BKA-inhibited structure (colored by domain, as in A–C) and CATR-inhibited structure (PDB: 4C9H, colored gray). (G) CATR binding induces a bend of H2 because K108 on H2 of ScAac2 (equivalent to R100 in TtAac) interacts electrostatically with the two sulfate groups of CATR. Displacement of the cytoplasmic end of H2 induces a similar displacement of H3. (H) Superposition of the crystal structure of BKA-inhibited TtAac (gray cartoon) and the uninhibited m-state model (colored by domain as in A–C). The uninhibited m-state model was generated by applying symmetry restraints between the domains, while treating them as rigid bodies. BKA induces a displacement of the matrix side of domain 1. The cytoplasmic side of the carrier is unaffected. (I) Comparison of BKA-inhibited and uninhibited m-state models suggests that the domain 1 displacement is due to the steric bulk of the inhibitor, combined with electrostatic repulsion between one of the BKA carboxyl groups and E37 on H1. Domain 3 has been removed to provide a clearer view of the inhibitor. See also Figures S3, S4, and S7.

Comparison of the structures of the three domains in BKA- and CATR-inhibited carriers (Figures 6A–6C) reveals that ∼80% of the domain structure is conserved between the two states, which we call the core element, comprising the odd-numbered, matrix, linker, and one-third of the even-numbered helices. In particular, there are no major changes to the kink angle around the proline residues of the signature motif, consistent with the notion that the kink is stabilized by interactions within the domain (Ruprecht et al., 2014). The proposal that the conformational change may involve these proline residues acting as hinges during transport, with the odd-numbered helices straightening during the transition to the m-state (Pebay-Peyroula et al., 2003), is not supported by the current structure. There is, however, a significant and unexpected movement of the C-terminal region of the even-numbered helices in all three domains toward the central axis from the c- to the m-state. Thus, the domains do not move as single rigid bodies and are more dynamic than originally anticipated (Ruprecht et al., 2014). In the m-state, the even-numbered helices kink at R88 (H2), G192 (H4), and R287 (H6), which are key amino acids predicted to be involved in substrate-binding, called the contact points of the substrate-binding site (Kunji and Robinson, 2006, Robinson and Kunji, 2006). The inward movement of these helical regions (comprising the even-numbered helices from the contact point to the C-terminal end), which we call the gate elements, contributes to bringing the residues of the cytoplasmic network together. Conformational change from the c- to m-state must involve rigid-body rotations of the core elements to open the central cavity to the mitochondrial matrix and inward movements of the gate elements, closing the central cavity to the intermembrane space.

### Inhibitor Binding Is Accompanied by Induced Fit

It has been proposed that the inhibitors CATR and BKA induce structural perturbations of the c- and m-states, because the inhibitors have larger molecular volumes than the substrates and maximize their interactions with the protein, thus increasing their binding affinity (Klingenberg, 2008). The inhibited states have therefore been called abortive states, which are not transport cycle intermediates. Comparing the structures binding either inhibitor allows us to investigate the structural consequences of inhibition.

We initially analyzed the domain structures. For the CATR-inhibited carriers, while most of the domain structure is conserved, there are noticeable deviations in the positions of the C-terminal end of H2 and N-terminal end of H3 (Figure 6D). The structures of the individual domains in the BKA-inhibited state superpose well, indicating that the inhibitor has not perturbed the structures of the domains themselves (Figure 6E).

To understand the consequences of CATR binding further, we have taken the core and gate elements from the BKA-inhibited structure and superposed them on the CATR-inhibited structure (Figure 6F). Core elements 1 and 3 (from domains 1 and 3) align very well, with root-mean-square deviations (RMSDs) of 0.97 and 1.16 Å for backbone atoms, respectively (Figures 6A and 6C). However, core element 2 does not align well, with an RMSD of 2.25 Å for backbone atoms (Figure 6B). Fitting core element 2 with outlier rejection revealed that while most of the structure fits well, there is a deviation at the N-terminal end of H3, due to change in the kink angle at the Pro of the signature motif (from 76° in the CATR-inhibited state to 43.0° in the BKA-inhibited state). When aligning the gate elements, H4 and H6 aligned well (backbone RMSDs of 0.95 and 0.85 Å, respectively), but H2 did not (backbone RMSD of 1.76 Å). Closer inspection found that this misalignment is due to the helix of the BKA-inhibited structure being straight, whereas that of the CATR-inhibited structure is kinked at Phe107 in CATR-inhibited ScAac2 (Phe99 in TtAac). This kink is caused by Lys108 interacting with the two sulfate groups of CATR, pulling H2 down and bringing the C-terminal end close to the end of H3, which is consequently displaced as well (Figure 6G). The deviations in the positions of H2 and H3 observed here are consistent with those seen when comparing the CATR-inhibited domains alone (Figure 6D). Therefore, the action of CATR is 2-fold—first, it acts as a competitive inhibitor by binding to the substrate-binding site, and second, it induces a structural change in H2 and H3 that locks the carrier in an abortive state.

**Figure S7:**
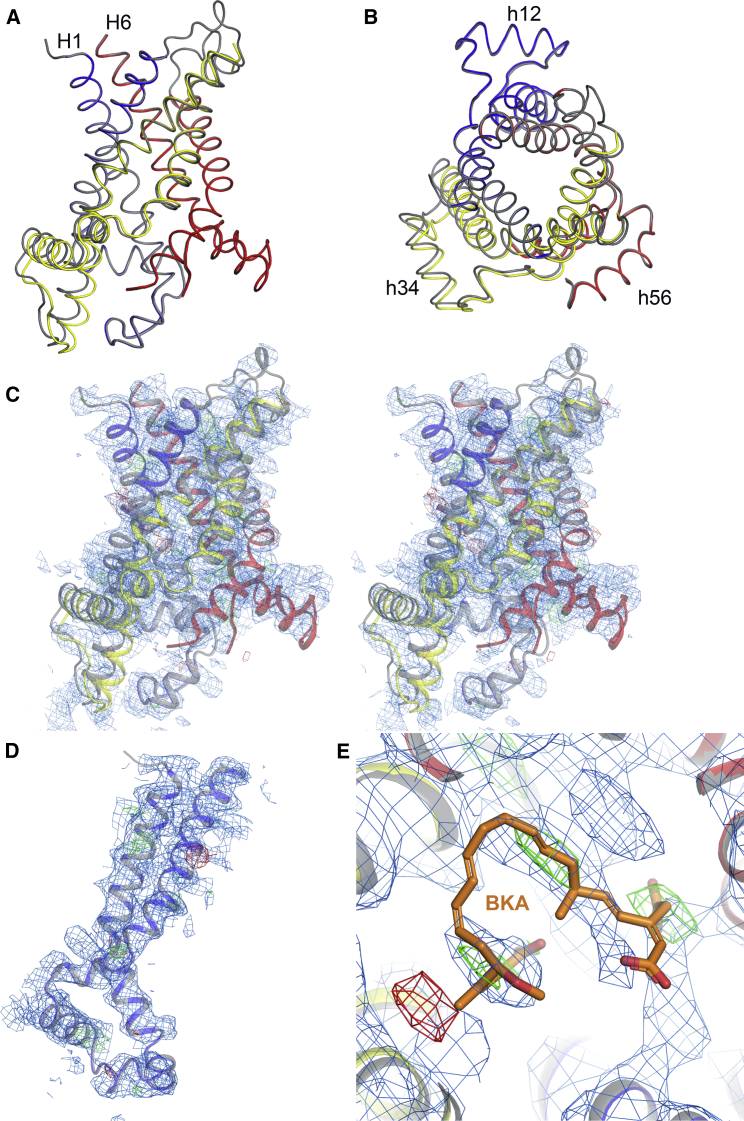
Comparison of BKA-Inhibited M-State Structures from Crystals Grown with and without Nanobody, Related to Figure 6 (A) Superposition of m-state carrier structures from crystals grown with nanobody (*P*3_2_21 space group, gray tube) and without nanobody (*P*2_1_2_1_2_1_ space group, tube with domain 1, blue; domain 2, yellow; domain 3, red), viewed from the membrane. The structures superimpose with 0.6 Å RMSD over 1096 atoms, showing that presence of the nanobody, different detergent and crystallization conditions, and different crystal packings have not perturbed the structure of the BKA-inhibited state. (B) The same superposition as (A), viewed from the cytoplasmic side of the membrane. (C) Stereo-view of electron density maps for the *P*2_1_2_1_2_1_ crystal form, following molecular replacement (using individual domains from the *P*3_2_21 crystal) and rigid-body refinement in AutoBuster. The *P*3_2_21 structure is shown as a gray cartoon, with the *P*2_1_2_1_2_1_ colored by domain. (D) Electron density for domain 1. (E) Partial density for BKA, not included in the molecular replacement search models, gives confidence in the quality of the molecular replacement phases. In (C)-(E), the 2*m*Fo-*D*Fc electron density map, contoured at 1 times the root mean square electron density (σ), is shown as a blue mesh, and the *m*Fo-*D*Fc electron density map is shown contoured at +3σ (green mesh) and −3σ (red mesh).

A noticeable difference between the BKA- and CATR-inhibited states is that the CATR-inhibited state is highly symmetrical, in agreement with the presence of three homologous sequence repeats, whereas the BKA-inhibited state is 3-fold pseudosymmetric at the cytoplasmic side but somewhat asymmetrical at the matrix side. To investigate the cause, we symmetrized the BKA-inhibited structure, while treating the domains as rigid bodies (Figure 6H), in agreement with our analysis of the domain structures (Figure 6E). Comparing the two structures, the positions of domains 2 and 3 are similar, but domain 1 is displaced toward the matrix side. In agreement, the cardiolipin-binding site between domains 2 and 3 is maintained in the BKA-inhibited structure, with good electron density present for the lipid (cdl 802), but the sites between domains 1 and 3 and domains 1 and 2 are disrupted (Figures 1 and S3). To exclude the unlikely possibility that the domain displacement is a consequence of crystal packing interactions or the nanobody interacting with H1 (Figure S2), we solved the structure of the BKA-inhibited carrier from a crystal grown without the nanobody, which diffracted anisotropically to 3.6 Å resolution (Table S1, TtAac crystal). Although the crystal diffracts to lower resolution than the TtAac-Nb crystal, it confirms that the structure of BKA-inhibited TtAac is not significantly affected by nanobody binding (Figure S7). The displacement of domain 1 is therefore likely to be due to inhibitor binding, leading to an abortive m-state. Closer inspection shows two likely molecular reasons for the abortive state. First, E37 in the symmetrized carrier lies in close proximity to negatively charged groups of BKA, so electrostatic repulsion may drive domain displacement. Second, the molecular volume of the inhibitor (443 Å^3^) is substantially greater than ATP (354 Å^3^), hence steric repulsion may also play a role (Figure 6I).

This comparison shows that the inhibitors perturb the structure of the protein, in agreement with earlier proposals. Both inhibitors occupy the positions where the substrates bind by mimicking the biophysical properties rather than the exact chemistry of the substrates and by forming multiple interactions that prevent the substrate competing for binding. Carboxylate groups of the inhibitors mimic the phosphate groups of the natural substrates. Furthermore, the binding itself distorts the structure of this highly dynamic protein, locking it in an abortive state that cannot undergo the required conformational changes for transport.

## Discussion

The available structures have revealed the conformational changes undergone by the domains. Further insight requires considering these domain changes in the context of the whole protein. A morph between the CATR- and BKA-inhibited states does not provide a plausible mechanism, because of the absence of an occluded state and because of clefts appearing in the lipid-facing part of the carrier. Having gained insight into the nature of inhibitor-induced distortions, we can use models of the uninhibited proteins to propose a plausible transport mechanism. Because CATR and BKA bind to different states of the protein, we generated a model of the uninhibited c-state from the superposition of the core and gate elements of the BKA-inhibited structure with the CATR-inhibited structure (Figure 6F). The uninhibited m-state model is the symmetrized model from the BKA-inhibited structure (Figure 6H). This seems a more reasonable approach than modeling the uninhibited m-state from elements of the CATR-inhibited structure, due to the CATR-induced kink in H2 and displacement of H3.

**Figure 7:**
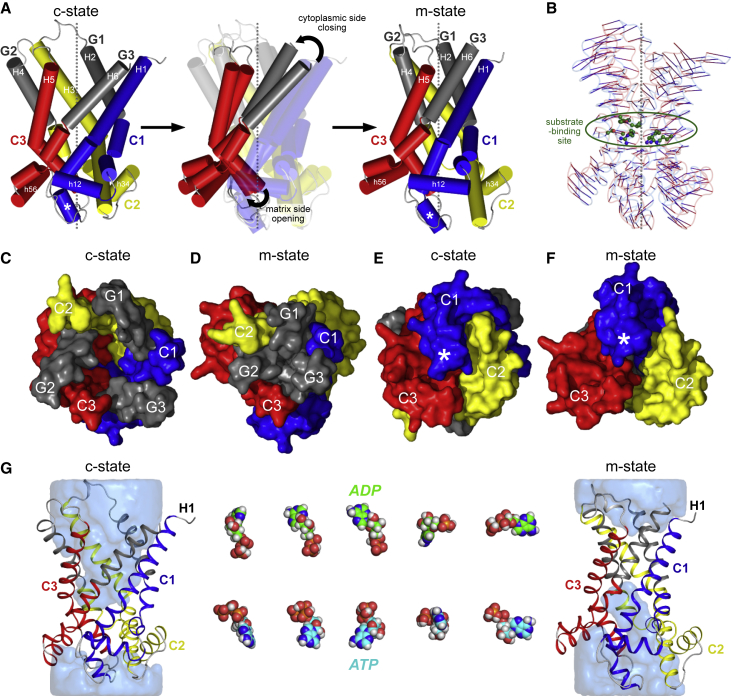
Proposed Transport Mechanism of the Mitochondrial ADP/ATP Carrier (A) Conformational changes between models of the uninhibited c- and m-states viewed laterally from the membrane, with helices shown as cylinders. The three-fold pseudosymmetry axis is shown as a dotted gray line. Conformational changes between c- and m-states can be described as a rotation of the core elements, coupled with an inward movement of the gate elements. Core elements 1, 2, and 3 are colored by domain in blue, yellow, and red, respectively, and the gate elements are colored gray. The asterisk marks the extra turn of α-helix on the matrix end of H1, seen in fungal ADP/ATP carriers. (B) Conformational changes between c- and m-states use the substrate-binding site as a fulcrum. Alignment of models of the uninhibited c- (blue) and m-states (red), with lines connecting equivalent Cα positions, generated by RAPIDO (Mosca and Schneider, 2008). Substrate-binding site residues are shown in green ball-and-sticks. The 3-fold pseudosymmetry axis is shown as a dotted gray line. (C–F) Models of the uninhibited c-state and m-states viewed from the cytoplasmic side of the inner mitochondrial membrane, (C) and (D) respectively, and from the mitochondrial matrix, (E) and (F) respectively, colored and labeled as in (A). Loop regions between H2-H3 and H4-H5 have been omitted to provide a clearer view of the core and gate elements. (G) Accessibility of the substrate-binding site in the uninhibited carrier models. The carrier is shown as a cartoon colored as in (A). The blue semi-transparent surface shows the internal cavities. ATP and ADP are shown in sphere representation in different views. See also Video S1.

Superposition of the uninhibited c- and m-states allows us to propose how the domain motions affect the overall conformation of the carrier, leading to opening and closing of the substrate-binding site to either side of the membrane. The core elements have a rocking motion of ∼15°, which is essential for opening and closing of the matrix side of the carrier (Figure 7A). The gate elements rotate toward or away from the central pseudosymmetry axis, respectively closing or opening access to the substrate-binding site from the intermembrane space (Figure 7A), with the contact points of the binding site acting as pivots (Kunji and Robinson, 2006, Robinson and Kunji, 2006). The conformational changes are small around the substrate-binding site but become progressively larger toward either side of the membrane (Figure 7B), indicating that the substrate-binding site acts as a fulcrum. Because the same substrate-binding site is accessible from both c- and m-states, and ADP and ATP are chemically related, the same process can apply in either direction across the membrane, explaining the known equimolar exchange activity. Atoms move predominantly parallel to the membrane, with no significant movement perpendicular, consistent with the transmembrane helices moving within the plane of the lipid bilayer. We speculate that engagement of the contact point residues with the substrate, leading to tighter interactions, drives the inward movement of the gate elements and formation of the cytoplasmic network. Concurrently, the cytoplasmic sides of the core elements also move inward, causing the core elements to rock and to open the matrix side. The involvement of all three contact points in substrate-binding makes it likely that the movements of the core and gate elements are symmetrical and simultaneous in all three domains, which also prevents clashes or gaps appearing in the protein structure. These movements lead to a coupled closing of the cytoplasmic side and an opening of the matrix side in the transition from c- to m-states, consistent with an alternating-access transport mechanism (Figures 7C–7G). The fungal ADP/ATP carriers have an extra turn of α-helix on the matrix end of H1, which is a loop in the bovine carrier. This extra turn slides out of the translocation pathway when the carrier transitions from the c- to the m-state, allowing sufficient access to the binding site (Figure 7G).

The cavity leading to the substrate binding site in the uninhibited m-state model is narrower than that of the c-state model (Figures 7C, 7F, and 7G). Access of substrate to the m-state binding site is likely to be facilitated by thermal energy driving rotamer changes, changing the size of the entry. However, the size difference between the cavities in the two states might reflect the biophysical requirements for transport of negatively charged nucleotides in the presence of a membrane potential. Import of ADP, which has three negative charges, is opposed by the membrane potential. Therefore, the carrier has a large water-filled cavity in the c-state to shield the substrate from the influence of the membrane potential until it is bound to residues of the substrate binding site. The negatively charged phosphate groups are neutralized by interactions with the three positively charged residues of the binding site (Kunji and Robinson, 2006), facilitating ADP import. The export of ATP, which has four negative charges, is stimulated by the membrane potential, which is maximal when shielding by water is minimal, explaining the relatively small cavity in the matrix state. Once ATP is bound to the central substrate binding site, a net negative charge remains, which helps to drive export.

A morph between the uninhibited c- and m-states (Video S1) demonstrates these proposals and passes through an occluded state, in which the substrate-binding site is inaccessible from either side of the membrane, which is essential for maintenance of the proton motive force across the inner mitochondrial membrane. The structure of the BKA-inhibited carrier provides the first insight into the structure of the m-state. It shows that the ADP/ATP carrier functions as a monomer (Bamber et al., 2007, Kunji and Crichton, 2010, Kunji and Harding, 2003), because the conformational changes alter the overall molecular shape profoundly, precluding stable interactions between carriers. The carrier has a single substrate-binding site that is alternately accessible from either side of the membrane, in agreement with inhibitor (Klingenberg, 1979) and sequence analysis (Robinson et al., 2008). The structure demonstrates the formation of the cytoplasmic salt bridge network in the m-state, showing that formation and disruption of the cytoplasmic and matrix salt bridge networks are key features of the transport cycle (King et al., 2016, Robinson et al., 2008, Ruprecht et al., 2014). It has also explained all of the conserved sequence features of mitochondrial carriers, including the role of bulky hydrophobic residues in forming the cytoplasmic gate and the role of the GxxxG and πxxxπ motifs in the dynamic conformational changes of the inter-helical interfaces during the transport cycle. Because these sequence features are conserved, it is likely that the proposed mechanism holds for all mitochondrial carriers, which constitute the largest solute carrier family in humans. The mechanism is unique among transport proteins, as it involves coupled rotations of three domains, with each domain undergoing two separate rotations, leading to alternating access from opposite sides of the membrane to the substrate-binding site. Together with the CATR-inhibited structures, this structure provides a framework for further biophysical and structural analysis of the intermediary states of the transport cycle, which will elucidate how substrates drive the conformational changes and how pathological mutations affect transport activity.

Video S1. Conformational Changes of the Mitochondrial ADP/ATP Carrier between C- and M-States, Related to Figure 7 This movie illustrates a morph between models of the uninhibited c-state and m-state. The domains of the carrier are colored separately: domain 1 (blue), domain 2 (yellow), domain 3 (red). The morph illustrates the conformational transition between c-state, with the central cavity open to the mitochondrial intermembrane space, and m-state, with the cavity open to the matrix side. The transition between states proceeds via an occluded state, in which the central substrate-binding site is inaccessible from either side of the membrane. Residues of the GxxxG motif, the πxxxπ motif and the cytoplasmic gate are shown as spheres scaled to van der Waals radii with magenta, green and orange carbons, respectively. Key functional elements are shown in ball-and-stick representation: substrate-binding site (green carbons) and matrix and cytoplasmic salt bridge networks (blue carbons for positively-charged residues, red carbons for negatively-charged residues). The lipid bilayer shown at the start of the video is a POPC lipid bilayer from https://heller.userweb.mwn.de/membrane (Heller et al., 1993).

## STAR★Methods

### Key Resources Table

**Table:** 

REAGENT or RESOURCE	SOURCE	IDENTIFIER
**Bacterial and Virus Strains**		

*E. coli* strain WK6 (su-)	ATCC	ATCC number:47078
*Lactococcus lactis* strain NZ9000	MoBiTec GmbH	Cat# VS-ELS09000-01

**Chemicals, Peptides, and Recombinant Proteins**		

Dodecyl-β-D-maltoside	Glycon Biochemicals GmbH	Cat#D97002
Decyl maltose neopentyl glycol (10-MNG)	Anatrace	Cat#NG322
HEGA-10	Anatrace	Cat#H110
N-[4-(7-diethylamino-4-methyl-3-coumarinyl)phenyl] maleimide (CPM)	Sigma Aldrich	Cat#C1484
Bongkrekic acid (BKA)	Sigma Aldrich	Cat#B6179
Complete Mini EDTA-free protease inhibitor tablets	Roche	Cat#05056489001
Nickel Sepharose (High Performance)	GE Healthcare	Cat#17526802
Nickel-NTA Superflow	QIAGEN	Cat#30430
Sephadex G75	GE Healthcare	Cat#17005001
Factor Xa protease	NEB	Cat#P8010L
Tetraoleoyl cardiolipin (TOCL)	Avanti Polar Lipid	Cat#710335
1,2-dioleoyl-*sn*-glycero-3-phosphocholine	Avanti Polar Lipid	Cat#850375
PFO cryo oil	Sigma Aldrich	Cat#317926
LV cryo oil	Mitegen	Cat#LVCO-1

**Critical Commercial Assays**		

Bicinchoninic acid (BCA) Protein assay kit	ThermoFisher Scientific	Cat#23227

**Deposited Data**		

Structure of yeast mitochondrial ADP/ATP carrier isoform 2 inhibited by carboxyatractyloside	Ruprecht et al., 2014	PDB: 4C9H
Nanobody targeting human Vsig4 in Spacegroup C2	Wen et al., 2017	PDB: 5IMK
BKA-inhibited TtAac-Nb complex structure	This paper	PDB: 6GCI
CPM and transport data	This paper	https://doi.org/10.17632/x4xdfh6gt2.1

**Experimental Models: Organisms/Strains**		

*Saccharomyces cerevisiae* W303-1B	ATCC	ATCC number:201238
*Saccharomyces cerevisiae* WB-12	Hashimoto et al., 1999; A gift from Dr H. Terada, Tokyo University of Science	N/A

**Oligonucleotides**		

See Table S2	This paper	N/A

**Recombinant DNA**		

Modified pYES3 vector	Bamber et al., 2006, King et al., 2016	pYES-pMIR2-AAC2
pNZ8048 vector N.B. pNZ8148 is a commercially available updated version of this vector	MoBiTec GmbH	Cat# VS-ELV00200-01
pMESy4 vector	GenBank	KF415192.1

**Software and Algorithms**		

XDS	Kabsch, 2010	http://xds.mpimf-heidelberg.mpg.de
AIMLESS	Evans and Murshudov, 2013	http://www.ccp4.ac.uk/html/aimless.html
AutoPROC	Vonrhein et al., 2011	http://www.globalphasing.com/autoproc/
STARANISO	Tickle et al., 2018	http://www.globalphasing.com/staraniso/manual/
Phenix	Adams et al., 2010	http://www.phenix-online.org
PHASER	McCoy et al., 2007	http://www.phaser.cimr.cam.ac.uk/index.php/Phaser_Crystallographic_Software
Parrot	Cowtan, 2010	http://www.ccp4.ac.uk/html/parrot.html
Coot	Emsley et al., 2010	https://www2.mrc-lmb.cam.ac.uk/personal/pemsley/coot/
Buster	Smart et al., 2012	http://www.globalphasing.com/buster/
Hollow	N/A	http://hollow.sourceforge.net
PyMOL	Schrödinger	https://pymol.org/2/
Molprobity	Chen et al., 2010	http://molprobity.biochem.duke.edu
UCSF Chimera	Pettersen et al., 2004	https://www.cgl.ucsf.edu/chimera/
Modeler 9.19	Sali and Blundell, 1993	https://salilab.org/modeller/

**Other**		

Proteus 1-Step Batch Mini Spin columns	Generon	Cat#GEN-1SBM-40
PD10 column	GE Healthcare	Cat#17043501
Amicon Ultra 50K centrifugal concentrators	Merck Millipore	Cat#UFC505024

### Contact for Reagent and Resource Sharing

Further information and requests for resources and reagents should be directed to and will be fulfilled by the Lead Contact, Edmund R.S. Kunji (ek@mrc-mbu.cam.ac.uk).

### Experimental Model and Subject Details

Wild-type ADP/ATP carriers for biophysical and structural studies were expressed in *Saccharomyces cerevisiae* strain WB-12 (MAT*α ade2-1 trp1-1 ura3-1 can1-100 aac1::LEU2 aac2::HIS3*), and grown in YEPG at 30°C. Mutant ADP/ATP carriers for biophysical studies were expressed in *Saccharomyces cerevisiae* strain W303-1B (*MAT*α *leu2-3,112 trp1-1 can1-100 ura3-1 ade2-1 his3-11,15*), and grown in YEPG + 0.1% glucose at 30°C. For transport assays, wild-type and mutant proteins were expressed in *Lactococcus lactis*, using the expression vector pNZ8048 and a nisin A-inducible promoter, and grown in M17 media supplemented with 1% (w/v) glucose and 5 μg/ml chloramphenicol. Nanobodies were expressed in *E. coli* strain WK6 (su-), grown in Terrific Broth at 28°C to an OD_600_ of 0.7-1.2 and induced with 1 mM isopropyl-β-D-thiogalactoside.

The project involved the generation of camelid antibodies for which llamas need to be immunized and blood samples collected. This is all done in compliance with both the European legislation (EU directive 2010/63/EC) and the Belgian Royal Decree of 29 May 2013 concerning the protection of laboratory animals with the exception that the animals are not specifically bred for such use. There are no breeders yet that do this. The llamas are housed in a center (farm) which is licensed by the Belgian competent authorities (accreditation number LA 1700601) and all staff involved is appropriately trained. The animals are very well taken care of and have plentiful access to food, drink and movement. The welfare of the llamas is being supervised by an accredited veterinarian. The immunization and blood collection of the llamas has been approved by the authorized local Animal Ethics Review board. The approval reference number is 13-601-3 and is valid until 30/09/2021.

### Method Details

#### Thermostability analysis of wild-type and mutant ADP/ATP carriers

Wild-type and mutant ADP/ATP carriers were expressed and purified using established procedures (King et al., 2016). The gene for the ADP/ATP carrier from *Thermothelomyces thermophila* (TtAac), encoding residues 8-311, was engineered with an upstream N-terminal His_8_ tag and Factor Xa protease cleavage site, and ligated into a modified pYES3 vector. Mutants of TtAac for biophysical characterization were produced by overlap-extension PCR. Expression vectors were electroporated into *Saccharomyces cerevisiae* strain W303-1B. Positive transformants were selected on Sc-Trp plates. Cells containing the desired construct were grown in 5 L of YEPG + 0.1% glucose at 30°C for 24 h, or in 50 L of YPG medium in an Applikon 140 Pilot System with an eZ controller. Mitochondria were prepared and proteins purified as described in King et al. (2016), but with 2% dodecyl-β-D-maltoside (Glycon Biochemicals GmbH) being used for solubilization of mitochondria. Thermostability data were obtained by using the thiol-reactive fluorophore N-[4-(7-diethylamino-4-methyl-3-coumarinyl)phenyl] maleimide (CPM) (Alexandrov et al., 2008), as described previously (King et al., 2016).

#### Transport assays of wild-type and mutant ADP/ATP carriers

For transport assays, wild-type and mutant proteins were expressed in *Lactococcus lactis*, using the expression vector pNZ8048 and a nisin A-inducible promoter. Following expression, membranes were isolated and fused with liposomes, and transport rates determined by measuring uptake of ^14^C-labeled ADP. Full details of these methods have been described previously (King et al., 2016).

#### Expression and purification of BKA-inhibited ADP/ATP carriers

The construct for the thermostabilized (Q302K mutation) ADP/ATP carrier from *Thermothelomyces thermophila* (TtAac), encoding residues 8-311, engineered with an upstream N-terminal His_8_ tag and Factor Xa protease cleavage site, was produced in a modified pYES3 vector as described previously (King et al., 2016). For purification of carrier-nanobody complexes, a modified construct was produced by PCR, encoding a Q302K mutation and MetSer upstream of residues 4-315 of the carrier, but without the His_8_ tag and Factor Xa site. The expression vectors were transformed by electroporation into *Saccharomyces cerevisiae* strain WB-12 (MAT*α ade2-1 trp1-1 ura3-1 can1-100 aac1::LEU2 aac2::HIS3*) (Hashimoto et al., 1999). Transformants were selected initially on Sc-Trp plates, and then on YEPG plates. For large scale fermentations, a 2-L pre-culture was used to inoculate 100 L of YEPG medium in an Applikon 140 Pilot System with an eZ controller. Cells were grown at 30°C for 72 h and harvested by centrifugation (4,000*g*, 20 min, 4°C). Mitochondria were prepared by disrupting cells with a bead mill (Dyno-Mill Multilab, Willy A. Bachofen AG Maschinenfabrik, Switzerland), as described (Thangaratnarajah et al., 2014). The total mitochondrial protein concentration was adjusted to 20 mg/mL with 0.1M MES pH 6.5, 10% glycerol. Mitochondria were flash frozen in liquid nitrogen, and stored at −80°C before use.

BKA-inhibited His-tagged TtAac was purified starting from 1 g of mitochondria. Carriers were inhibited with 2 nmol bongkrekic acid (BKA, Sigma Aldrich) per mg mitochondrial protein, supplemented with 250 μM ADP (50 mL final volume), and two Roche Complete Mini EDTA-free protease inhibitor tablets at room temperature for 30 min. Subsequent steps were performed at 4°C. Mitochondria were pelleted by centrifugation (48,000*g*, 15 min, 4°C), the supernatants removed, and the pellets resuspended in an equal volume of 100 mM Tris pH 7.4, 10% glycerol. Mitochondria were solubilized in 2% decyl maltose neopentyl glycol (10-MNG, Anatrace) solution, with 20 mM imidazole, 150 mM NaCl, 5 μM BKA and two Roche Complete Mini EDTA-free protease inhibitor tablets, in a final volume of 90 mL, for 1 h at 4°C. The solubilizate was clarified by centrifugation (200,000*g*, 30 min, 4°C) before loading onto a 1-mL Ni Sepharose column (GE Healthcare, High Performance). The column was washed with 40 mL of buffer A (10 mM Tris pH 7.4, 150 mM NaCl, 20 mM imidazole, 0.1 mg/mL tetraoleoyl cardiolipin (Avanti Polar Lipid), 0.02% 10-MNG, 5 μM BKA), followed by 25 mL of buffer B (10 mM Tris pH 7.4, 50 mM NaCl, 0.05 mg/mL tetraoleoyl cardiolipin, 0.02% 10-MNG, 10 μM BKA), at a flow rate of 3 mL/min. The Ni Sepharose was recovered as a slurry (total volume ≈2 mL), supplemented with 10 mM CaCl_2_ and 60 μg Factor Xa protease (NEB), and incubated overnight at 10°C. The slurry was transferred into empty Proteus 1-Step Batch Mini Spin columns (Generon) and the protein was eluted from the resin by centrifugation (500*g*, 5 min, 4°C). Residual Ni Sepharose was removed by an additional centrifugation step (12,000*g*, 10 min, 4°C). The protein concentration was determined by BCA (Thermo Scientific), using bovine serum albumin as standard. Protein for crystallization experiments was concentrated to 2.5 mg/mL using centrifugal concentrators (Amicon Ultra 50 K).

#### Purification of nanobody targeting BKA-inhibited carrier

For nanobody generation, one llama (*Lama glama*) was immunized with BKA-inhibited thermostabilized TtAac, which had been reconstituted into 1,2-dioleoyl-*sn*-glycero-3-phosphocholine:tetraoleoyl cardiolipin (20:1 g/g ratio, Avanti Polar Lipid) liposomes at a 10:1 lipid:protein ratio (wt:wt), using established methods (Lee et al., 2015). A phage display library of nanobodies was prepared in the pMESy4 vector from peripheral blood lymphocytes as described (Pardon et al., 2014). Nanobodies were identified by selecting phages that bound to solid-phase immobilized proteoliposomes in the presence of BKA, and confirming lack of binding to CATR-inhibited protein reconstituted into proteoliposomes, or to empty liposomes. Six nanobody families were identified that specifically bound the BKA-inhibited protein, one of which included the nanobody used for crystallization and structure determination (nanobody CA9848). For purification of nanobody-carrier complexes, nanobody constructs were modified by introducing a Factor Xa cleavable C-terminal His_8_ tag via PCR. Nanobodies were expressed in the periplasm of *E. coli* strain WK6 (su-), following methods described previously (Pardon et al., 2014). Briefly, 1-L cultures in Terrific Broth were grown to an OD_600_ of 0.7-1.2 and induced with 1 mM isopropyl-β-D-thiogalactoside. Cells were harvested after overnight growth at 28°C, and periplasmic extract prepared using TES (Tris EDTA Sucrose) buffer. Nanobodies were purified from the periplasmic extract by Ni-NTA (QIAGEN) affinity chromatography.

#### Purification of BKA-inhibited TtAac-Nb complexes

Mitochondria (500-650 mg total protein) were inhibited with 2 nmol bongkrekic acid (BKA, Sigma Aldrich) per mg mitochondrial protein, supplemented with 20 μM ADP (final volume of 25 mL), at room temperature for 2 h. All subsequent steps were performed at 4°C. Mitochondria were pelleted by centrifugation (48,000*g*, 15 min, 4°C), the supernatants were removed, and the pellets were resuspended in an equal volume of Tris-buffered saline. Mitochondria were solubilized in 2% 10-MNG (Anatrace) solution, with 20 mM imidazole, 150 mM NaCl, 5 μM BKA and one Roche Complete Mini EDTA-free protease inhibitor tablet, in a final volume of 45 mL, for 1 h at 4°C. The solubilizate was clarified by centrifugation (200,000*g*, 30 min, 4°C). 3 mg purified His-tagged nanobody was added to the solubilizate and incubated for 30 min at 4°C, before adding Ni Sepharose (GE Healthcare, High Performance, 0.5 mL resin) and continuing the incubation overnight. The suspension was poured into an empty XK16 column (GE Healthcare) and packed by gravity flow. The column was washed with 15.5 mL of buffer C (10 mM Tris pH 7.4, 150 mM NaCl, 20 mM imidazole, 0.02 mg/mL tetraoleoyl cardiolipin (Avanti Polar Lipid), 0.35% HEGA-10 (Anatrace), 5 μM BKA), followed by 17 mL of buffer D (10 mM Tris pH 7.4, 150 mM NaCl, 10 mM imidazole, 10 mM CaCl_2_, 0.35% HEGA-10, 10 μM BKA), at a flow rate of 0.4 mL/min. The slurry was recovered (volume ≈1.3 mL), supplemented with 30 μg Factor Xa protease and incubated at 10°C for 2 h. The slurry was transferred into empty Proteus 1-Step Batch Mini Spin columns (Generon) and protein was eluted from the resin by centrifugation (500*g*, 5 min, 4°C). The protein was concentrated to a 0.5 mL volume using centrifugal concentrators (Amicon Ultra 50 K) and applied to a 4-mL Sephadex G75 column (GE Healthcare) packed in an empty PD10 column (GE Healthcare) and equilibrated with 10 mM Tris pH 7.4, 50 mM NaCl, 0.35% HEGA-10, 10 μM BKA. The protein was applied and eluted by gravity flow, with the TtAac-Nb complex eluting in volume fractions between 0.5-1.5 mL. The protein was concentrated to A_280_ ≈8-10 using centrifugal concentrators (Amicon Ultra 50 K) and used in crystallization trials.

#### Crystallization

Crystals were grown using the sitting drop vapor diffusion technique. Crystallization plates were set up with drop ratios of 200 nL protein and 200 nL precipitating solution, or 500 nL of protein and 500 nL of precipitating solution, using a Mosquito robot (TTP Labtech). Crystals of TtAac were grown at 22°C using a precipitating solution of 90 mM HEPES pH 7.0, 1.8% 3-methyl-3-pentanol, 30% PEG600 and 0.5 mM dodecyl-β-D-maltoside (Glycon Biochemicals GmbH). Crystals were harvested from the drops and flash frozen in liquid nitrogen. Crystals used for structure determination of the TtAac-Nb complex came from two conditions setup at 4°C, either: 0.1M HEPES pH 7.5, 3% 3-methyl-3-pentanol, 18% PEG400; or 0.1M MES pH 6.5, 1% 3-methyl-3-pentanol, 22% PEG400. Crystallization plates were incubated at 10°C, and crystals appeared within 3 days. Crystals were cryo-protected using PFO (Sigma Aldrich) or LV (Mitegen) cryo oils, and flash frozen in liquid nitrogen.

#### Data collection and structure determination

Diffraction data for the TtAac crystal were collected at 100 K at the European Synchrotron Radiation Facility beamline ID23-2 with a Mar 225 CCD detector using a 10 μm microfocus X-ray beam (0.8729 Å wavelength). Diffraction data for the TtAac-Nb crystals were collected at 100 K at Diamond Light Source beamline I24 with a Pilatus 6M detector using a 9x6 μm microfocus X-ray beam (0.9686 Å wavelength). Data were collected using helical data collection methods. Datasets were indexed, integrated, scaled and merged using the programs XDS (Kabsch, 2010) and AIMLESS (Evans and Murshudov, 2013), as implemented in AutoPROC (Vonrhein et al., 2011). Due to the anisotropic diffraction from the crystals, datasets were ellipitically truncated and corrected using STARANISO (Tickle et al., 2018), as implemented in AutoPROC. Data collection statistics are shown in Table S1.

Structure determination of the TtAac-Nb complex was initiated using a crystal that diffracted anisotropically to 3.9 Å along 0.894 *a^∗^* - 0.447 *b^∗^*, 3.9 Å along *b^∗^* and 3.1 Å along *c^∗^*. Analysis by phenix.xtriage (Adams et al., 2010) showed that the crystal was untwinned. Phases were determined by molecular replacement in PHASER (McCoy et al., 2007), by searching sequentially with a homology model of the Nb (based on PDB: 5IMK; translation function Z score (TFZ) = 8.1, log likelihood gain (LLG) = 43), then with a homology model for domain 1 (residues 16-45 and 61-90 based on CATR-inhibited *Saccharomyces cerevisiae* ScAac2, PDB: 4C9H chain A; TFZ = 10.8, LLG = 113). Domain 2 (residues 116-152 and 165-208 based on PDB: 4C9H chain A) could be placed by molecular replacement, but it was clear from the electron density maps, and from maps produced after density modification with Parrot (Cowtan, 2010), that the C-terminal end of H4 was in a different position. This region was rebuilt, and molecular replacement with domain 2 repeated (TFZ = 16.6, LLG = 297). At this stage, electron density maps allowed the manual placement and rebuilding of domain 3 (residues 226-252 and 262-294). Density features, such as the extra turn of helix on the matrix side of H1, as observed in the yeast c-state structures (Ruprecht et al., 2014), helped to confirm the arrangement of domains. Iterative cycles of model building in Coot (Emsley et al., 2010) and refinement in Buster (Bricogne et al., 2017) were carried out. B-factor sharpened maps were used to help confirm side-chain positions. Refinement with Buster used LSSR restraints and grouped B-factors (2 per residue, for main chain and side chain). In the early stages of model building and refinement, LSSR restraints were from the nanobody homology model, and a carrier homology model based upon PDB: 4C9H chain A. In later stages, LSSR restraints were based on models of the TtAac-Nb complex that had been refined with phenix.refine (Afonine et al., 2012) using secondary structure restraints. B-factor refinement included TLS parameterization (1 TLS group per chain). Diffraction data were collected from a crystal diffracting to higher resolution (statistics in Table S1), also untwinned, and model building and refinement were continued using these data, maintaining the flagged reflections from the earlier dataset for calculating R_free_. BKA was modeled into positive *m*Fo-*D*Fc difference electron density present in the central cavity. Several factors were considered during the modeling of BKA: (1) the stereochemistry of the ligand, combined with minimal geometrical distortions following refinement, (2) minimal clashes with protein residues, (3) maximal number of protein-ligand interactions, (4) maximal cross-correlation with the 2*m*Fo-*D*Fc density map. Refinement statistics are shown in Table S1. The final model consists of residues 11-252 and 257-306 of TtAac, residues 1 to 124 of the nanobody, a molecule of BKA and of PEG, and partially modeled cardiolipins in the asymmetric unit. The cardiolipin between domains 2 and 3 has good density. However, density for cardiolipins between domains 1 and 2 and domains 1 and 3 is of poorer quality, due to the BKA-induced displacement of domain 1 making it impossible for cardiolipins to bridge the distance between the even-numbered transmembrane and matrix helices. Both these cardiolipins have been modeled as partial lipids at both positions. It is likely that these cardiolipins interact predominantly with either the even-numbered transmembrane or matrix helix, stochastically throughout the crystal. Residues M250-S252 and K257 of the carrier lie in very weak electron density in this crystal, but their position is supported by good density in the earlier crystal.

Phases for the TtAac structure (without nanobody) were determined by molecular replacement with PHASER (McCoy et al., 2007), searching with carrier domains from the TtAac-Nb model (domain 1: residues 13-107, domain 2: residues 116-211, and domain 3: 220-306; TFZ = 17.2, LLG = 378). Rigid-body refinement was carried out in Buster (Bricogne et al., 2017), with each domain defined as a rigid-body, reaching R = 36.2%, R_free_ = 37.1%. The resulting electron density maps showed density for features not included in the model (loop regions, BKA and cardiolipin phosphates, Figure S7). The carrier structure superposes very well with that solved from the TtAac-Nb crystals (RMSD of 0.6 Å over 1096 matched atoms). Splitting domain 1 into smaller rigid sub-domains, or performing jelly-body refinement in REFMAC, did not change the structure.

Comparisons of the TtAac BKA-inhibited m-state structure with the ScAac2 CATR-inhibited c-state shown in the figures used PDB: 4C9H chain A. The program HOLLOW (http://hollow.sourceforge.net) was used to generate the blue semi-transparent surfaces shown in Figures 2A, 2B, and 7G. Protein structure figures were generated in PyMOL. The polder OMIT map (Liebschner et al., 2017) for BKA was calculated after perturbing the TtAac-Nb model by removing the BKA coordinates and subjecting the model to coordinate and group B-factor refinement in phenix.refine. The coordinates of BKA were added back prior to polder map calculation, to define the region for bulk solvent exclusion while being excluded from map calculation.

#### Models of the uninhibited c- and m-states

The model of the uninhibited c-state of TtAac was generated by superposing the core and gate elements from the BKA-inhibited TtAac structure onto the structure of CATR-inhibited ScAac2p (PDB: 4C9H chain A) in PyMOL. Superposition of the backbones of core domains 1 and 3, and of even-numbered helices 4 and 6 could be accomplished without outlier rejection. Superposition of the backbones of core domain 2 and even-numbered helix 2 required outlier rejection and refinement, as implemented in the align command. As discussed in the manuscript, these differences are attributable to CATR-induced protein distortions. The superposed elements of the TtAac crystal structure provided an initial model for the uninhibited c-state, which was completed by loop building based upon the available crystal structures. The structure was energy-minimized in Chimera (Pettersen et al., 2004), rotamer outliers were corrected in PyMOL, and the geometry was checked by Molprobity (Chen et al., 2010).

To create a model of the uninhibited m-state of TtAac, the structure of the BKA-inhibited carrier was initially symmetrized using Modeler 9.19 (Sali and Blundell, 1993), applying a symmetry restraint with weight 2 between the Cα atoms of domains 1 and 2 and between the Cα atoms of the respective symmetry-related residues of domains 2 and 3. Symmetrization corrected the displacement of domain 1, resulting in domain 1/domain 2 and domain 1/domain 3 interfaces with suitable geometry for cardiolipin-binding. The symmetrised models were used as a template for placing the domains from the crystal structure, treating the domains as rigid bodies. This provided an initial model for the uninhibited m-state, which was completed by loop building based upon the available crystal structures. The geometry was checked by Molprobity (Chen et al., 2010), rotamer outliers were corrected in PyMOL, and the structure was energy-minimized in UCSF Chimera (Pettersen et al., 2004) to reduce clashes.

The models of the uninhibited states were aligned using RAPIDO (Mosca and Schneider, 2008), prior to generating a morph in Chimera (Pettersen et al., 2004).

### Quantification and Statistical Analyses

For CPM-based thermostability analysis, the apparent melting temperature was calculated from the first derivative of the thermal denaturation profile, and represented by the average and standard deviation of three technical repeats (Figures S6E and S6F) or six repeats (n = 3 with two biological repeats, for Figure 3C). Significance was assessed by a two-tailed Student’s t test assuming equal variance, with *P value*s indicated as follows: p > 0.05, not-significant (NS); p > 0.01, ^∗^; p > 0.001, ^∗∗^; p > 0.0001, ^∗∗∗^; p > 0.00001, ^∗∗∗∗^. The null hypothesis is that the mutation has no effect upon the melting temperature of the BKA-inhibited protein.

For the transport studies, the residual transport rate is represented by the average and standard deviation of four technical repeats, with each mutant having its own wild-type comparison (Figures 3E and S5A). Significance was assessed by a two-tailed Student’s t test assuming unequal variance, with *P value*s indicated as follows: p > 0.05, not-significant (NS); p > 0.01, ^∗^; p > 0.001, ^∗∗^; p > 0.0001, ^∗∗∗^; p > 0.00001, ^∗∗∗∗^. The null hypothesis is that the mutation has no effect upon the transport rate.

### Data and Software Availability

Coordinates, structure factor amplitudes and Fourier map coefficients for the nanobody-bound BKA-inhibited mitochondrial ADP/ATP carrier (TtAac-Nb) have been deposited in the Protein Data Bank under accession number PDB: 6GCI. The structure factor file includes structure factors for the *P*2_1_2_1_2_1_ TtAac crystal as a second data block. CPM and transport assay data have been deposited as a Mendeley dataset (https://doi.org/10.17632/x4xdfh6gt2.1).
