# Supplementary material for: The Molecular Mechanism of Transport by the Mitochondrial ADP/ATP Carrier
Source: Cell. 2019 Jan 24;176(3):435–447.e15. doi: 10.1016/j.cell.2018.11.025 (PMC6349463; doi:10.1016/j.cell.2018.11.025)
Supplement: Document S1. Tables S1 and S2 [file mmc1.pdf]

Cell, Volume *176*

Supplemental Information

The Molecular Mechanism of Transport by the Mitochondrial ADP/ATP Carrier

Jonathan J. Ruprecht, Martin S. King, Thomas Zögg, Antoniya A. Aleksandrova, Els Pardon, Paul G. Crichton, Jan Steyaert, and Edmund R.S. Kunji

Table S1. Data collection, refinement and validation statistics. Related to STAR methods.

	TtAac-Nb complex†	TtAac‡
**Data collection***		
Space group	$P3_221$	$P2_12_12_1$
Cell dimensions		
a, *b*, *c* (Å)	75.65, 75.65, 295.48	76.59, 77.26, 96.54
α, β, γ (°)	90.0, 90.0, 120.0	90.0, 90.0, 90.0
Resolution (Å)	29.35-3.30 (3.43-3.30)	96.54-3.85 (4.24-3.85)
R_{merge}	0.063 (0.688)	0.103 (0.960)
$I / \sigma I$	12.1 (2.3)	9.1 (1.4)
Completeness (spherical, %)	85.1 (40.3)	56.9 (13.6)
Completeness (ellipsoidal, %)	91.9 (88.8)	88.5 (73.2)
Redundancy	4.7 (4.7)	3.5 (3.5)
CC(1/2)	1.000 (0.823)	0.999 (0.558)
**Refinement**		
Resolution (Å)	29.35-3.30	
No. reflections work set/test set	13203/628	
R_{work}/R_{free} (Buster)	25.2/28.3	
No. atoms		
Protein	3064	
Ligand/ion	182	
Water	0	
Mean *B*-factors (Å^2)		
Protein	110.8	
BKA/CDL/PEG	80.4/136.4/89.6	
R.m.s. deviations		
Bond lengths (Å)	0.008	
Bond angles (°)	0.87	
Ramachandran favoured (%)	96.8	
Ramachandran outliers (%)	0.0	
Molprobity score	1.47	

*Values in parentheses are for highest-resolution shell.

†Diffraction data collected from one crystal, which diffracted anisotropically to 3.51 Å along 0.894 *a**- 0.447 *b**, 3.51 Å along *b** and 3.04 Å along *c**.

‡Diffraction data collected from one crystal, which diffracted anisotropically to 3.61 Å along *a**, 5.6 Å along *b**, and 4.4 Å along *c**.

Table S2. DNA oligonucleotide sequences. Related to STAR methods.

Oligonucleotide	Sequence 5’-3’
TtAac no tag forward	CATGACATGTCTAAACAAGAAACTAAAATTTTAGGAATGCCACCCTTCGTG
TtAac no tag reverse	CTAGCTCGAGCTATCATTAACCAGATCCACCTTTAAAAGCTTTGCCGAATAGTAGAATTTGC
Nb Xa-His8 forward	TCACAAGCTTAAGGAGACAGTACA
Nb Xa-His8 reverse	CTAGTCCGGATTATTAATGGTGATGGTGATGGTGGTGATGTTCGCTGGTCGCGGTACGACCTTCGATCGCACGGTCTGAGGAGACGGTGACCTG
Q302K reverse	CTCGAGTCTAGATCATTTGCCGAATAGTAGAATTTTCAATTGATCGTATATGGACAACAC
K30A forward	CTGCAGCAGTTAGTGCTACAGCTGC
K30A reverse	GCAGCTGTAGCACTAACTGCTGCAG
R88A forward	GCCAACGTTATAGCTTATTTTCCAACCCAG
R88A reverse	CTGGGTTGGAAAATAAGCTATAACGTTGGC
N96A forward	TCCAACCCAGGCATTGGCTTTTGCTTTTAG
N96A reverse	CTAAAAGCAAAAGCCAATGCCTGGGTTGGA
R100A forward	GAACTTTGCTTTTGCTGATAAGTTTAAGGC
R100A reverse	GCCTTAAACTTATCAGCAAAAGCAAAGTTC
G192A forward	GGTCCATCCGTGGCCGCTATTGTTGTTTACAGA
G192A reverse	TCTGTAAACAACAATAGCGGCCACGGATGGACC

Oligonucleotide	Sequence 5’-3’
TtAac no tag forward	CATGACATGTCTAAACAAGAAACTAAAATTTTAGGAATGCCACCCTTCGTG
TtAac no tag reverse	CTAGCTCGAGCTATCATTAACCAGATCCACCTTTAAAAGCTTTGCCGAATAGTAGAATTTGC
Nb Xa-His8 forward	TCACAAGCTTAAGGAGACAGTACA
Nb Xa-His8 reverse	CTAGTCCGGATTATTAATGGTGATGGTGATGGTGGTGATGTTCGCTGGTCGCGGTACGACCTTCGATCGCACGGTCTGAGGAGACGGTGACCTG
Q302K reverse	CTCGAGTCTAGATCATTTGCCGAATAGTAGAATTTTCAATTGATCGTATATGGACAACAC
K30A forward	CTGCAGCAGTTAGTGCTACAGCTGC
K30A reverse	GCAGCTGTAGCACTAACTGCTGCAG
R88A forward	GCCAACGTTATAGCTTATTTTCCAACCCAG
R88A reverse	CTGGGTTGGAAAATAAGCTATAACGTTGGC
N96A forward	TCCAACCCAGGCATTGGCTTTTGCTTTTAG
N96A reverse	CTAAAAGCAAAAGCCAATGCCTGGGTTGGA
R100A forward	GAACTTTGCTTTTGCTGATAAGTTTAAGGC
R100A reverse	GCCTTAAACTTATCAGCAAAAGCAAAGTTC
G192A forward	GGTCCATCCGTGGCCGCTATTGTTGTTTACAGA
G192A reverse	TCTGTAAACAACAATAGCGGCCACGGATGGACC
I193A forward	CCATCCGTGGCCGGTGCTGTTGTTTACAGAGGT
I193A reverse	ACCTCTGTAAACAACAGCACCGGCCACGGATGG
